# Southern rice black-streaked dwarf virus induces incomplete autophagy for persistence in gut epithelial cells of its vector insect

**DOI:** 10.1371/journal.ppat.1011134

**Published:** 2023-01-27

**Authors:** Lu Zhang, Wenwen Liu, Nan Wu, Hui Wang, Zhongkai Zhang, Yule Liu, Xifeng Wang

**Affiliations:** 1 State Key Laboratory for Biology of Plant Diseases and Insect Pests, Institute of Plant Protection, Chinese Academy of Agricultural Sciences, Beijing, China; 2 Beijing Key Laboratory of Maize DNA Fingerprinting and Molecular Breeding, Maize Research Institute, Beijing Academy of Agriculture and Forestry Sciences, Beijing, China; 3 Biotechnology and Germplasm Resources Institute, Yunnan Key Laboratory of Agricultural Biotechnology, Yunnan Academy of Agricultural Sciences, Kunming, China; 4 MOE Key Laboratory of Bioinformatics and Center for Plant Biology, School of Life Sciences, Tsinghua University, Beijing, China; Agriculture and Agri-Food Canada, CANADA

## Abstract

Autophagy plays an important role in virus infection of the host, because viral components and particles can be degraded by the host’s autophagy and some viruses may be able to hijack and subvert autophagy for its benefit. However, details on the mechanisms that govern autophagy for immunity against viral infections or benefit viral survival remain largely unknown. Plant reoviruses such as southern rice black-streaked dwarf virus (SRBSDV), which seriously threaten crop yield, are only transmitted by vector insects. Here, we report a novel mechanism by which SRBSDV induces incomplete autophagy by blocking autophagosome-lysosome fusion, resulting in viral accumulation in gut epithelial cells of its vector, white-backed planthopper (*Sogatella furcifera*). SRBSDV infection leads to stimulation of the c-Jun N-terminal kinase (JNK) signaling pathway, which further activates autophagy. Mature and assembling virions were found close to the edge7 of the outer membrane of autophagosomes. Inhibition autophagy leads to the decrease of autophagosomes, which resulting in impaired maturation of virions and the decrease of virus titer, whereas activation of autophagy facilitated virus titer. Further, SRBSDV inhibited fusion of autophagosomes and lysosomes by interacting with lysosomal-associated membrane protein 1 (LAMP1) using viral P10. Thus, SRBSDV not only avoids being degrading by lysosomes, but also further hijacks these non-fusing autophagosomes for its subsistence. Our findings reveal a novel mechanism of reovirus persistence, which can explain why SRBSDV can be acquired and transmitted rapidly by its insect vector.

## Author summary

Reoviruses can cause serious harm to mammals and cereals in the world. Understanding their persistence mechanism can contribute to control strategies. Here, we reveal that a plant reovirus, SRBSDV can activate the JNK signaling pathway through interacting with integrin β3 in the plasma membrane, upregulate expression of autophagy-related genes, and increase autophagosome numbers. Once inside the insect cell, SRBSDV hijacks autophagosome for accumulation, further escape degradation by inhibiting fusion between autophagosomes and lysosomes by the interaction of major outer capsid protein with LAMP1. This new mechanism explains why SRBSDV can survive in and be efficiently transmitted by a particular vector insect species and will help to design novel strategies to prevent virus transmission.

## Introduction

Southern rice black-streaked dwarf virus (SRBSDV) was firstly detected in 2001 in Guangdong Province, China and recognized as a novel virus in family *Reoviridae* in 2008, mainly based on genomic and transmission characteristics [1,2]. During the 2000s, it spread widely in China, Vietnam, Korea, Japan and Thailand, becoming one of the most important rice pathogens in East and Southeast Asia [3–5]. Five other reoviruses are known to infect rice: rice black-streaked dwarf virus (RBSDV), rice dwarf virus, rice gall dwarf virus (RGDV), rice bunchy stunt virus and rice ragged stunt virus [6,7]. Field epidemics of rice-infecting reoviruses largely coincide with the population density and transmission efficiency of their insect vectors because insect transmission is the only natural means for the spread of these viruses [8,9]. These viruses are persistently transmitted and propagated during infection of their insect vectors. Once rice reoviruses are ingested by the insects, they firstly infect a limited number of gut epithelial cells by binding to receptors on the cell membrane [7]. Then the viruses replicate in the infected cells, and the progeny virions spread to neighboring cells or directly into the hemocoel. Finally, the viruses enter the salivary glands via the circulating hemolymph and are horizontally transmitted to a plant as the insect feeds [7]. To survive in the insects, viruses must overcome multiple barriers and evade attack by the host immune system.

During infection, some viruses can also active autophagy in their insect vectors [10–12]. Autophagy is a highly evolved, conserved degradation process through lysosome in eukaryotes to maintain cell maturation and survival and homeostasis of the cellular environment by degrading senescent organelles and pathogenic microorganisms [13]. After initiation of autophagy, the membrane of pre-autophagosomes begins to extend outward, forming a phagocytic vesicle with a bilayer membrane that enfolds components in the cytoplasm that need to be degraded. As the phagocytic vesicles extend, the bilayer membrane eventually bends to form a double-membraned autophagosome [14]. The outer membrane of the autophagosome fuses with the membrane of the lysosome to enable entry of the autophagosomes into the lysosomal cavity, where the components are eventually degraded [15].

At present, more than 40 specific autophagy-related genes (*Atg*) have been identified as being involved in regulating autophagy [16]. During autophagy, Atg8 is usually cleaved to form soluble Atg8-I, which covalently binds to phosphatidylethanolamine to form Atg8-II on the outer membrane of the autophagosome. The appearance of Atg8-II marks the activation of autophagy pathways [17]. Two interconnected ubiquitin-like conjugation systems are involved in the formation of Atg8-II [18,19]. One system is activated by Atg7 and results in the covalent binding of Atg8 with Atg3. In the other system, Atg12 is constitutively conjugated to Atg5 by a sequential reaction involving Atg7 and Atg10 [20]. The Atg12-Atg5 conjugate has ligase-like activity to facilitate Atg8 conjugation to autophagic membranes [19].

Autophagy results from the activation of a mitogen-activated protein kinase signaling pathway (MAPK), such as the c-Jun N-terminal kinase (JNK) signaling pathway, and/or the inhibition of the mammalian target of rapamycin (mTOR) signaling pathway [21–23]. Several reports demonstrated that JNK can be activated by integrins when cells are attached to the extracellular matrix [24–26]. The JNK pathway is generally thought to induce autophagy by upregulating Beclin-1, which as a basic regulator of autophagy can activate autophagy. JNK phosphorylation will activate c-Jun and then enhance the transcriptional activity of Beclin-1 [27]. JNK can also phosphorylate Bcl-2 family proteins and prevent their interaction with Beclin-1; thus, the released Beclin-1 can activate autophagy [28,29]. Apart from phosphorylating proteins, JNK might also regulate autophagy by directly affecting expression of the target *Atg* genes. In oncogenic cells, activation of the JNK pathway leads to increased levels of Atg5, which enhances the levels of autophagy [30], but inhibition of JNK activity decreases Atg7 levels, which reduces the levels of autophagy [31,32].

Generally, autophagy can inhibit viral infection, restrict viral replication or regulate inflammatory response and promote antigen presentation [33]. For example, poliovirus infection can be restricted by initiating autophagous degradation of the viral RNA genome in the cytoplasm [34]. Rift valley fever virus can be degraded by lysosomes via autophagy [35]. Autophagy in the vector whitefly can be activated by tomato yellow leaf curl virus to defend the vector against viral infection and transmission [11]. On the other hand, although autophagy defends host cells against exogenous viruses, the persisting viruses can also evolve various strategies to escape or inhibit multiple steps of the autophagy pathway for their survival [33]. Herpes simplex virus encodes a neurovirulence factor that can interact with Beclin-1 to inhibit autophagy [36]. Some viral proteins can also block fusion between autophagosomes and lysosomes to inhibit the maturation of autolysosomes. For example, the viral phosphoprotein of parainfluenza virus type 3 binds to SNAP29 and inhibits its interaction with syntaxin17, which prevent the two proteins from mediating autophagosome-lysosome fusion [37]. For plant reovirus, RGDV can induce autophagy and utilize autophagosomes to carry virions and thus facilitate virus spread in the vector leafhopper (*Recilia dorsalis*) [10]. However, RBSDV has been reported to activate autophagy in its vector, the small brown planthopper (SBPH, *Laodelphax striatellus*), resulting in suppression of infection and thus virus accumulation [12].

In the present study to learn more about the mechanism(s) involved, we found that SRBSDV infection can also induce autophagy in its vector white-backed planthopper (WBPH, *Sogatella furcifera*), and the activation of autophagy contributed to virus survival because the virus used the autophagosome membrane as a scaffold for viral maturation and accumulation. Furthermore, the viral capsid protein P10 was found to inhibit autophagosome maturation to fusion with lysosomes by interacting with LAMP1. Our results revealed a novel mechanism by which the virus not only prevents its degradation by blocking the fusion of autophagosomes- lysosomes, but also hijacks these autophagosomes for its accumulation.

## Results

### SRBSDV can induce autophagy by activating the JNK signaling pathway in its insect vector

**Fig 1:**
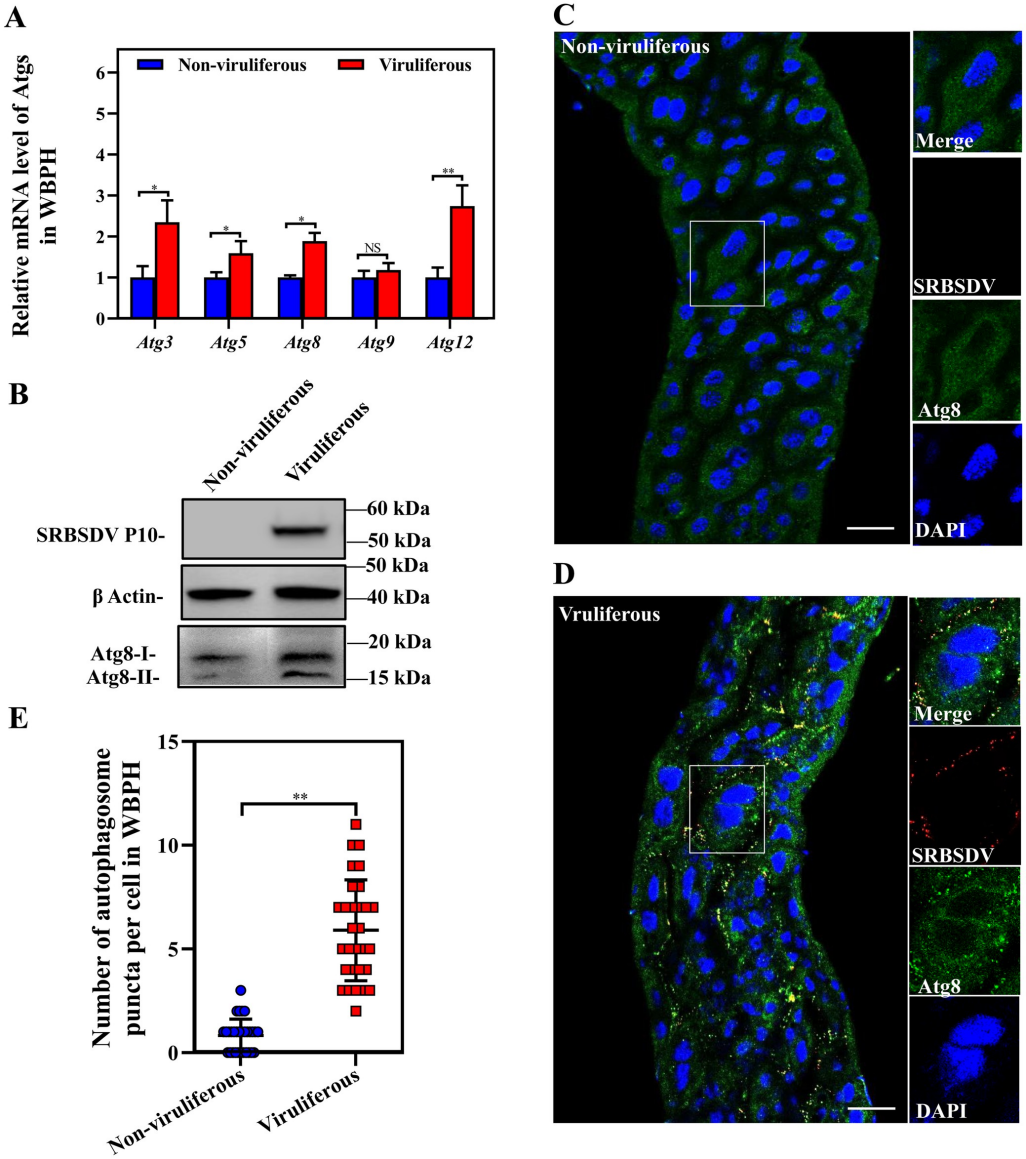
Assessment of autophagy levels in nonviruliferous and SRBSDV-viruliferous WBPH. (**A**) Relative mRNA levels for different *Atg* genes from nonviruliferous and viruliferous WBPHs as determined by RT-qPCR using total RNA. Total RNA was extracted from 30 insects in bulk for each group. (Means ± SEM of three independent experiments, *P < 0.05, **P < 0.01, Student’s *t* -test) (**B**) Western blots of Atg8-I and Atg8-II in nonviruliferous and viruliferous WBPHs determined by western blotting to show differences in autophagy. (**C**, **D**) LSCM of midguts of nonviruliferous and viruliferous WBPHs were respectively excised, then incubated with anti-Atg8 antibody (green) and DAPI (blue) and observed. Merged image is a closeup of area in white rectangle in left image. Scale bars, 20 μm. (**E**) Mean number of Atg8 puncta from 30 cells in each insect group was analyzed by Prism 8. (**P < 0.01, Student’s *t*-test).

We extracted total RNA from nonviruliferous or SRBSDV-viruliferous insects to quantify the relative mRNA levels of different *Atg* genes using RT-qPCR. The results showed that the relative mRNA levels of *Atg3*, *Atg5*, *Atg8* and *Atg12* but not *Atg9* increased significantly in SRBSDV-infected insects (Fig 1A). Total proteins from the viruliferous and the nonviruliferous insects were extracted and separated by SDS-PAGE gel electrophoresis. The western blots showed an obvious Atg8-II band in the viruliferous insects (Fig 1B).

Since the midgut epithelium is the initial infection site of SRBSDV, individual midguts were excised, incubated with anti-Atg8 antibody (green) and DAPI (blue), then observed with a laser scanning confocal microscope (LSCM). Fluorescence signals from Atg8 were distributed diffusely in the nonviruliferous insect midgut epithelial cells, but present as autophagosome puncta in the viruliferous insect midgut epithelial cells (Fig 1C and 1D). The average number of Atg8 puncta in each nonviruliferous cell was 0.833, which significantly differed from the number in a viruliferous cell (5.900) (Fig 1E).

**Fig 2:**
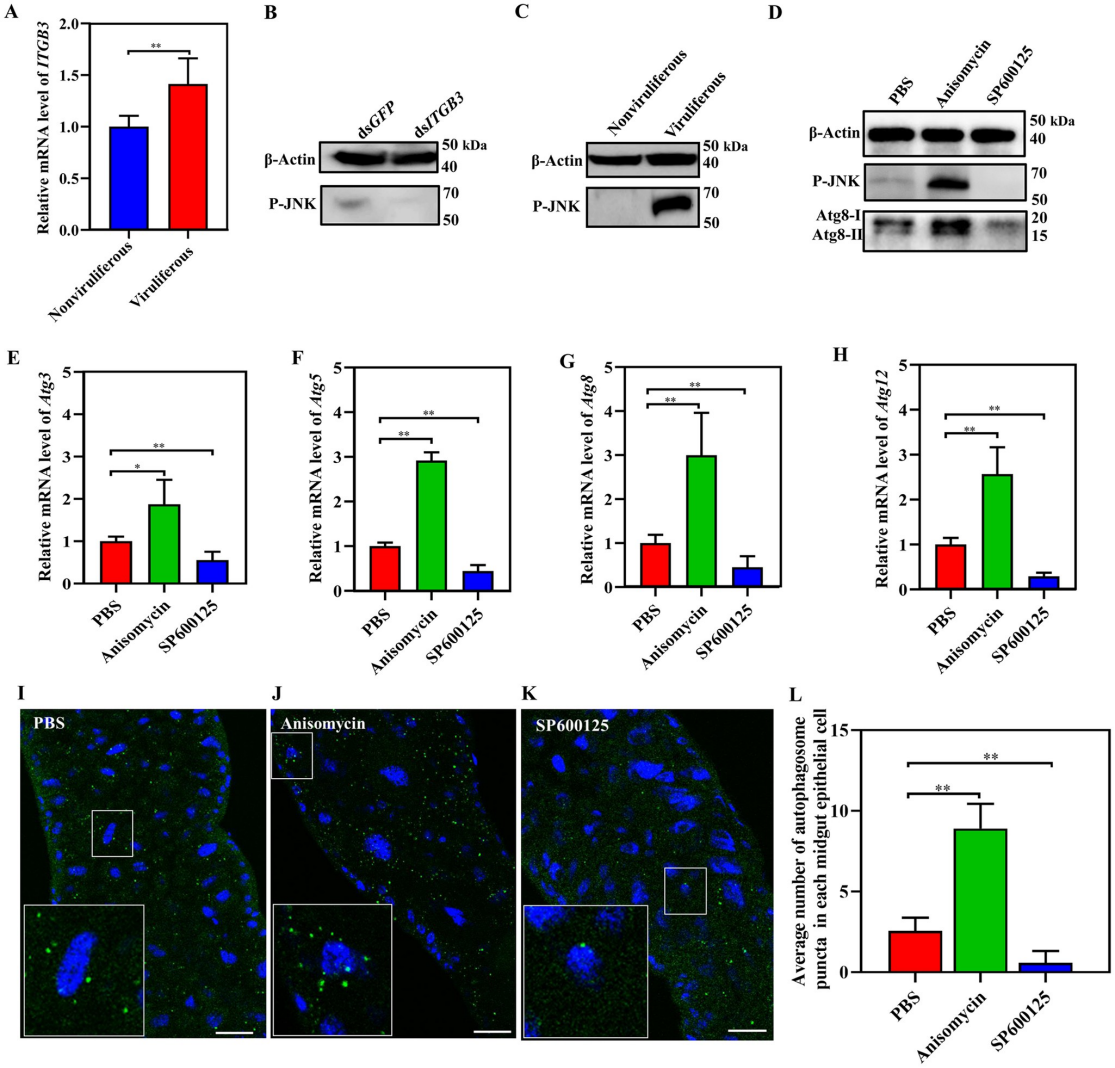
Activation of JNK signaling pathway promoted autophagy in WBPHs. (**A**) Relative mRNA level of *ITGB3* from nonviruliferous and viruliferous WBPHs as determined by RT-qPCR using total RNA. Total RNA was extracted from 30 insects in each group. (Means ± SEM of three independent experiments, *P < 0.05, **P < 0.01, Student’s *t*-test) (**B**) Western blot of P-JNK in total proteins from viruliferous WBPHs 2 d after injection with ds*GFP* or ds*ITGB3*. (**C**) Western blots of P-JNK from total proteins extracted from nonviruliferous and viruliferous WBPHs. β-Actin was the internal reference. (**D**) Western blots of P-JNK and Atg8 from nonviruliferous WBPHs after injection with PBS, JNK activator (anisomycin), or JNK inhibitor (SP600125) for 2 d, insects were starved. β-Actin was the internal reference. (**E**-**H**) RT-qPCR determination of relative mRNA levels of *Atg3*, *Atg5*, *Atg8* and *Atg12* after the three treatments. (Means ± SEM of three independent experiments, *P < 0.05, Student’s *t*-test). (**I**-**K**) LSCM of autophagosomal puncta in WBPH midgut epithelial cells. The midguts of the three groups were excised and incubated with anti-Atg8 antibody (green) and DAPI (blue). Scale bars, 20 μm. (**L**) Average number of autophagosome puncta in each midgut epithelial cell.

Our previous study identified a cell receptor protein (integrin β3, ITGB3) which interacts with SRBSDV P10 in yeast two-hybrid assays [38]. RT-qPCR revealed that the relative mRNA level of *ITGB3* increased after virus infection (Fig 2A). Integrins are thought to activate autophagy through JNK signaling pathway as indicated by the phosphorylation of JNK (phospho-JNK, P-JNK) [24–27]. Therefore, we injected insects with ds*ITGB3* and total proteins of insects were used to detect P-JNK by western blots. The results showed that the level of P-JNK decreased after interference with the expression of *ITGB3* gene (Fig 2B). To determine whether P-JNK activates autophagy, we extracted total proteins from nonviruliferous and viruliferous insects to detect by western blots and found that SRBSDV infection enhanced the level of P-JNK (Fig 2C). We then injected nonviruliferous insects with PBS, the JNK activator anisomycin, or the JNK inhibitor SP600125, then after 2 days, starved the insects for 2 h to induce the JNK pathway, only the treatment with anisomycin enhanced P-JNK and expression level of Atg8 (Fig 2D). RT-qPCR demonstrated that relative mRNA levels for *Atg3*, *Atg5*, *Atg8* and *Atg12* significantly increased after treatment with anisomycin, whereas the relative mRNA levels of *Atg3*, *Atg5*, *Atg8* and *Atg12* decreased after treatment with SP600125 (Fig 2E–2H). After the three treatments, the gut from the treated insects was excised and incubated with anti-Atg8 antibody (green) and DAPI (blue) and then observed with LSCM. The average numbers of autophagosome puncta in each midgut epithelial cell from these three groups were 2.567, 8.900, and 0.600. The results showed that the number of autophagosome puncta were higher after the JNK pathway was activated but lower after it was repressed compared with that of the PBS treated insects (Fig 2I–2L). We also injected insects with ds*ITGB3* during the process of virus entering in the midgut epithelial cells to detect relative mRNA levels of *Atg genes* by RT-qPCR. The results showed that interfering with *ITGB3* expression causes a significant decrease the relative mRNA level and protein expression level of *Atg*s (S1 Fig). These results confirmed that SRBSDV infection stimulated the JNK signaling pathway through virions binding to integrin β3 in the plasma membrane of epithelial cells. Then, the activation of JNK pathway upregulated expression of *Atg* genes which further activated autophagy.

### SRBSDV localized on the autophagosome membrane

**Fig 3:**
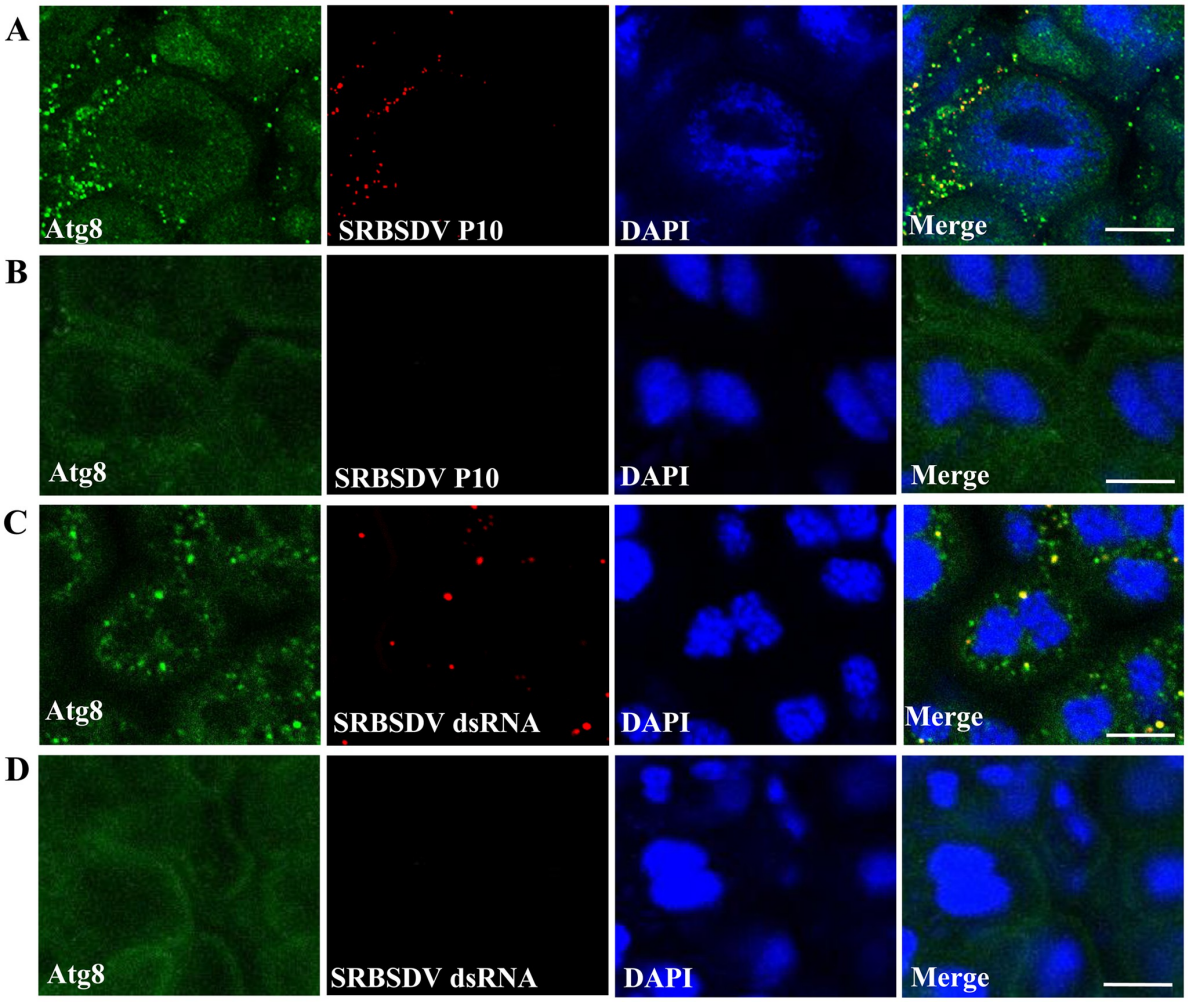
SRBSDV colocalized with autophagosomes in midgut epithelial cells. **(A, B)** Colocalization of autophagosome puncta (labeled by anti-Atg8 antibody, green) and SRBSDV capsids (labeled with anti-SRBSDV P10 antibody, red). **(C, D)** Colocalization of autophagosome puncta (labeled with anti-Atg8 antibody, green) and SRBSDV genomic RNAs (labeled with anti-double strand RNA antibody, red) in nonviruliferous and viruliferous. Scale bars, 10 μm.

**Fig 4:**
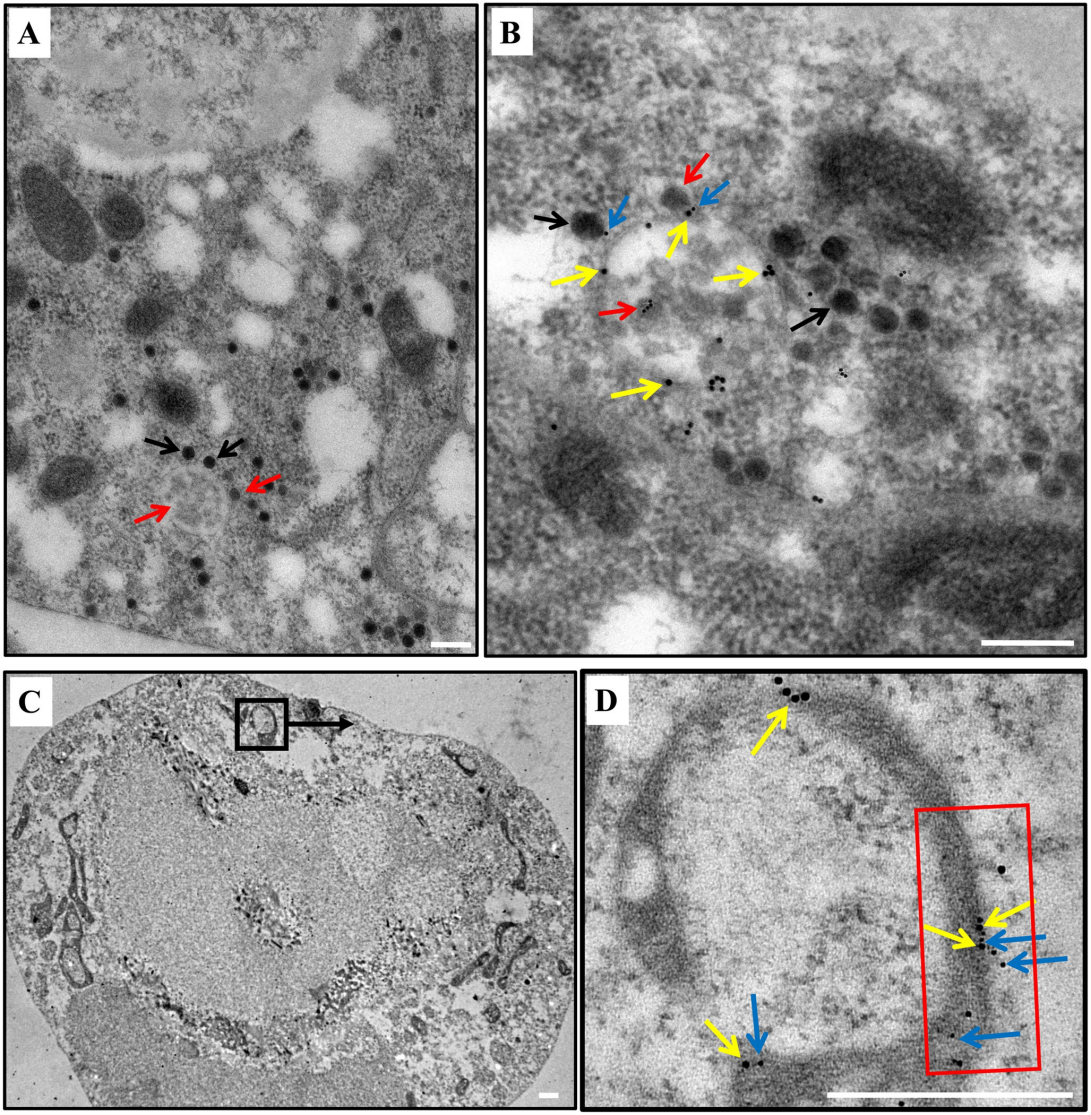
SRBSDV localized on the autophagosome membrane. (**A**) TEM of viruliferous WBPH midgut epithelial cells containing autophagosome-like double-membrane structures surrounded by immature and mature SRBSDV virions. Red arrow: immature SRBSDV virions; black arrow: mature SRBSDV virions. (**B**) iTEM of viruliferous WBPH midgut epithelial cells containing autophagosome structures surrounded by immature and mature SRBSDV virions. Red arrow: immature SRBSDV virions; black arrow: mature SRBSDV virions; blue arrows: SRBSDV (5 nm); yellow arrows: Atg8 (10 nm) Scale bars, 200 nm. **(C)** Colocalization of Atg8 and SRBSDV P10 on autophagosome membrane in cultured Sf9 cells. (**D**) Detail of area in black rectangle in (C). Blue arrows: P10 (5 nm); yellow arrows: Atg8 (10 nm); scale bars, 500 nm; red rectangle: double -membrane structure of autophagosome.

Interestingly, autophagosome puncta labeled by anti-Atg8 antibody (green) colocalized with SRBSDV capsids labeled by anti-SRBSDV P10 antibody (red) (Fig 3A and 3B) and with the SRBSDV genome labeled by anti-double strand RNA antibody (red) in viruliferous midgut epithelial cells (Fig 3C and 3D). This indicated that the capsid protein of SRBSDV and the viral genome dsRNA are present on the autophagosome. In parallel, observation by electron microscope transmission electron micrographs (TEM) and immune TEM (iTEM), the cytoplasm of the midgut epithelial cells of viruliferous insect had autophagosome structures that were surrounded by SRBSDV virions, but the nonviruliferous insect did not (Fig 4A and 4B, and S2 Fig). Autophagosomes contained only immature virions, while immature and mature SRBSDV virions were found around the edge of the outer layer of the membrane and some mature virions appeared to be released from the membrane (Fig 4A and 4B). These suggested that the virions accumulation on the autophagosome membrane. When SRBSDV P10 and Atg8 were expressed in cell lines of *Spodoptera frugiperda* (Sf9), then observed with immunoelectron microscopy, P10 and Atg8 were colocalized on the autophagosome membrane, indicating that the virus was located on the autophagosome membrane though its P10 binding to Atg8 (Fig 4C and 4D). These results suggest that the autophagosome membrane serves as a site for virions location and accumulation.

### Autophagy levels rose contributed to increase of virus titer

**Fig 5:**
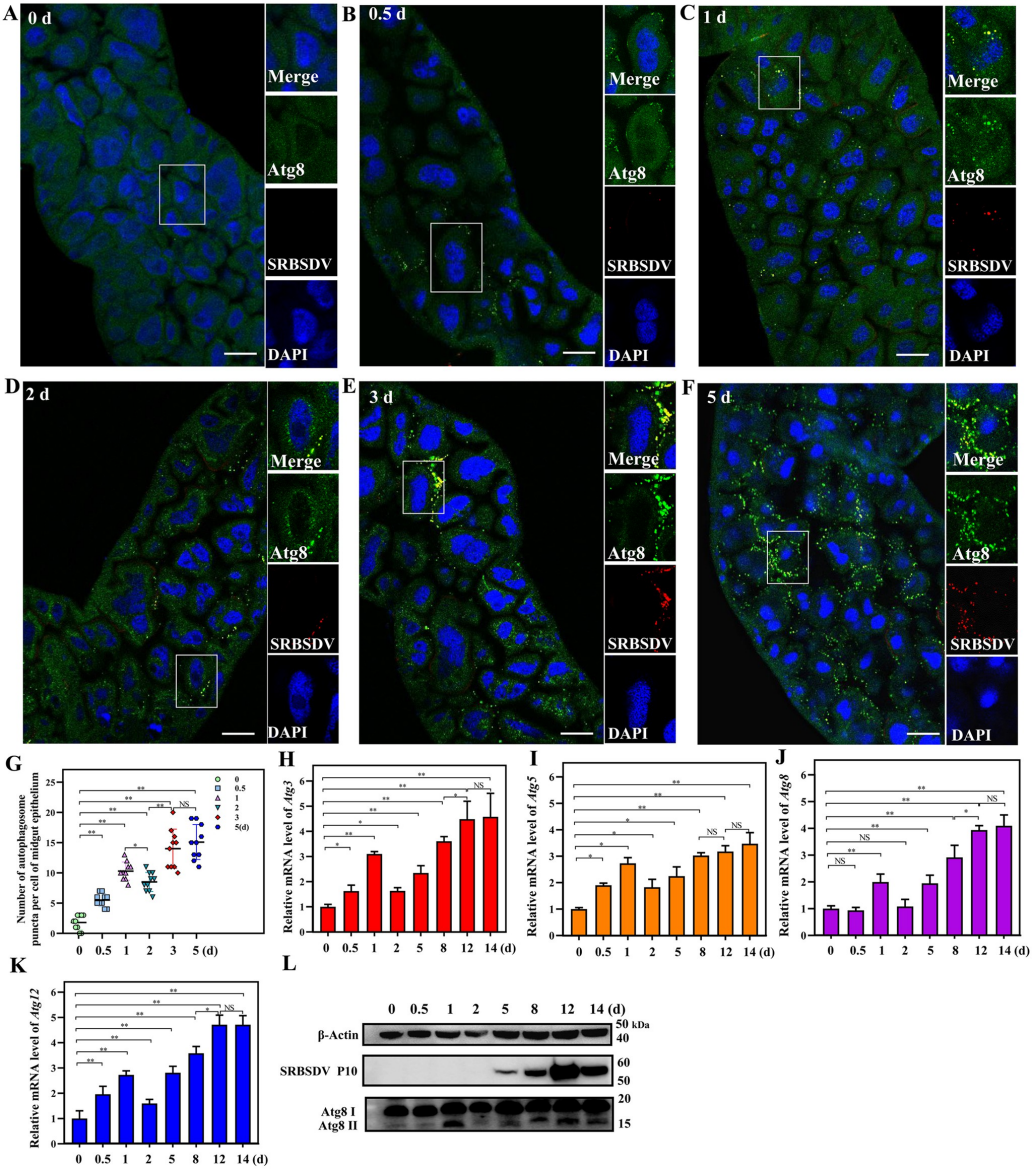
The level of autophagy increased as propagation of SRBSDV increased over time in viruliferous WBPHs. The number of autophagosome puncta was monitored over time during a 36-h acquisition access period (AAP) to explore the relationship between autophagy and propagation. (**A**-**F**) LSCM of 30 midguts of viruliferous WBPHs were excised at 0, 0.5, 1, 2, 3 and 5 d after a 36-h AAP, incubated with anti-Atg8 antibody (green), anti-SRBSDV antibody (red) and DAPI (blue) and observed with laser scanning confocal microscope. Scale bars, 10 μm. (**G**) Mean number of autophagosomal puncta in 10 midgut epithelial cells at 0, 0.5, 1, 2, 3 and 5 d after a 36-h AAP. (NS: not significant, *P < 0.05, **P < 0.01, Student’s *t*-test). (**H**-**K**) RT-qPCR determination of mRNA levels of *Atg3*, *Atg5*, *Atg8* and *Atg12* in total RNAs extracted at 0, 0.5, 1, 2, 5, 8, 12 and 14 d from 30 viruliferous WBPHs after a 36-h AAP. (Means ± SEM of three independent experiments, *P < 0.05, Student’s *t*-test). (**L**) Western blots to detect Atg8-I and II in total proteins extracted at 0, 0.5, 1, 2, 5, 8, 12 and 14 d from 30 viruliferous WBPHs after a 36-h AAP.

Because autophagy and virus propagation are dynamic processes, we monitored the number of autophagosomes over time during a 36-h acquisition access period (AAP) to explore the relationship between autophagy and virus propagation. Thirty midguts were respectively excised at 0, 0.5, 1, 2, 3 and 5 d, then incubated with anti-Atg8 antibody (green), anti-SRBSDV P10 antibody (red) and DAPI (blue). LSCM observation showed that SRBSDV fluorescence signals colocalized with autophagosome puncta in the midgut epithelial cells (Fig 5A–5F). The number of autophagosome puncta increased from 0 to 1 d, reached a small peak at 1 d, but had decreased rapidly by 2 d and then gradually increased from 2 d and until a plateau at 5 d in the midgut epithelial cells (Fig 5G). Then the total RNA from insects was extracted at 0, 0.5, 1, 2, 5, 8, 12 and 14 d after a 36-h AAP for RT-qPCR of *Atg* genes. The results showed that relative mRNA levels of *Atg3*, *Atg5*, *Atg8*, and *Atg12* also first increased to a small peak at 1 d, then decreased rapidly, then gradually increased over time until they plateaued (Fig 5H–5K). According to western blots, changes in Atg8-II levels were similar (Fig 5L) and increased, then plateaued as SRBSDV replicated. These results indicated that autophagy was activated immediately when SRBSDV initially invaded the epithelial cells but decreased after the virus entered the cells, then autophagy was further activated and sustained at high levels as the virus titer.

### Inhibition or activation of autophagy was closely related to virus titer and viral transmission efficiency of vector insects

**Fig 6:**
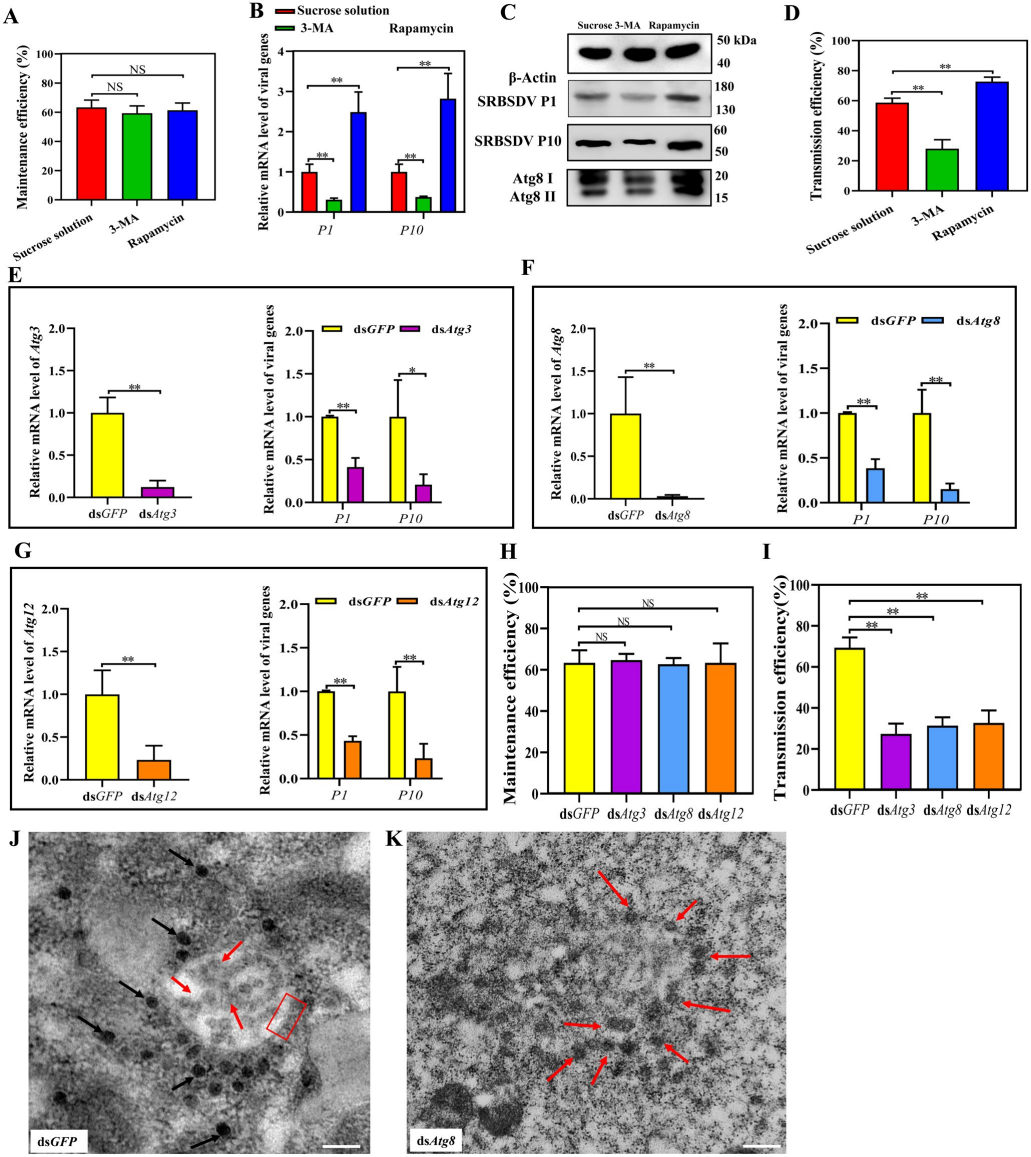
Autophagy inhibition repressed SRBSDV propagation; activation enhanced SRBSDV propagation. Two hundred WBPHs were fed 15% w/v sucrose solution (control), 1 μM 3-MA or 10 μM rapamycin for 24 h after a 36-h AAP and reared on rice seedlings for 2 d. After 4 d, 50 insects from each treatment were collected to determine the percentage of infected insects and mRNA level of P10, 50 insects were used for western blots to assess P10 levels, and 50 other insects were moved onto separate rice seedlings for 2 d to test virus transmission efficiency. (**A**) Percentage of infected WBPHs in the three treatment groups. (Means ± SEM of three independent experiments, Student’s *t*-test). (**B**) Relative mRNA levels for P1 and P10 in the three treatment groups. (Means ± SEM of three independent experiments, *P < 0.05, Student’s *t*-test) (**C**) Western blots of P1, P10 and Atg8 in the three treatment groups. β-Actin was the internal reference. (**D**) Transmission efficiency of viruliferous WBPHs after the three treatments. (Means ± SEM of three independent experiments, *P < 0.05, Student’s *t*-test). (**E**-**G**) Relative mRNA level of P1 and P10 after WBPHs were injected with ds*Atg3*, ds*Atg8* or ds*Atg12* (controls: ds*GFP*). (Means ± SEM of three independent experiments, *P < 0.05, Student’s *t*-test). (**H**) Percentage of infected WBPHs in the four dsRNA treatment groups. (Means ± SEM of three independent experiments). (**I**) Transmission efficiency of viruliferous WBPHs in the four dsRNA treatment groups. (Means ± SEM of three independent experiments, *P < 0.05, Student’s *t*-test). (**J**, **K**) TEM of midgut epithelial cells from ds*GFP-* or ds*Atg8*-injected viruliferous WBPHs. Red arrow: immature SRBSDV virions; black arrow: mature SRBSDV virions; red rectangle: bilayered-membrane structure of autophagosome. Scale bars, 200 nm.

To verify whether autophagy influences SRBSDV propagation in insects, 200 insects were respectively fed 15% w/v sucrose solution, 1 μM 3-MA or 10 μM rapamycin for 24 h after a 36-h AAP and reared on rice seedlings for 2 d. After 4 d, 50 insects from each treatment were collected to assess the percentage of infected insects and mRNA level of P10, and another 50 insects were used for western blots to assess P10 levels. The percentage of infected insects did not differ after autophagy was inhibited by 3-MA or activated by rapamycin (Fig 6A, S1 Table), but the mRNA level and protein level of P1 (viral RNA-dependent RNA polymerase) and P10 decreased after autophagy being inhibited but increased after autophagy being activated (Fig 6B and 6C), indicating that inhibition of autophagy repressed SRBSDV titer but activation of autophagy promoted SRBSDV titer.

Insects from these treatments were then assessed for their efficiency in transmitting the virus. From each treatment, 50 different insects were moved onto separate rice seedlings for 2 d. These insects were removed, and the seedlings were then grown in a greenhouse for 14 d, when total RNA of each plant was extracted to detect the *P10* gene by RT-PCR to calculate the SRBSDV transmission efficiency of each insect. Transmission efficiency was found to decrease by 30% for the 3-MA-treated insects, but increase by 14% for the rapamycin-treated insects (Fig 6D, S2 Table). These results demonstrated that the activation of autophagy in vector insects facilitated SRBSDV propagation and increased infection of plants.

For further verification of these findings, 100 insects that had a 36-h AAP and were reared on rice seedlings for 2 d were respectively injected with double-stranded (ds) *GFP*, ds*Atg3*, ds*Atg8* or ds*Atg12*. After 4 days, the percentage of infected insects and the relative mRNA levels of different *Atg* genes and P1 and P10 genes in the insects were determined. Compared with the ds*GFP*-injected group, the group injected with ds*Atg3*, ds*Atg8* or ds*Atg12* had lower levels of relative mRNA for P1 and P10 (Fig 6E–6G), but the percentage of infection did not differ significantly among the injection-treatment groups (Fig 6H, S3 Table). The transmission efficiency on rice plants of ds*Atg*3-, ds*Atg*8- and ds*Atg*12-inhibited insects was 42%, 38% and 36.6% lower, respectively, than that of the ds*GFP*-injected insects (Fig 6I, S4 Table). TEM results showed that, after the interference by ds*Atg8*, autophagosomes did not form and the virus particles were mostly present as empty shells (Fig 6J and 6K). Thus, blocking the formation of autophagosomes impaired maturation of the virions.

### The autophagosome membrane served as a site for P10 aggregation

**Fig 7:**
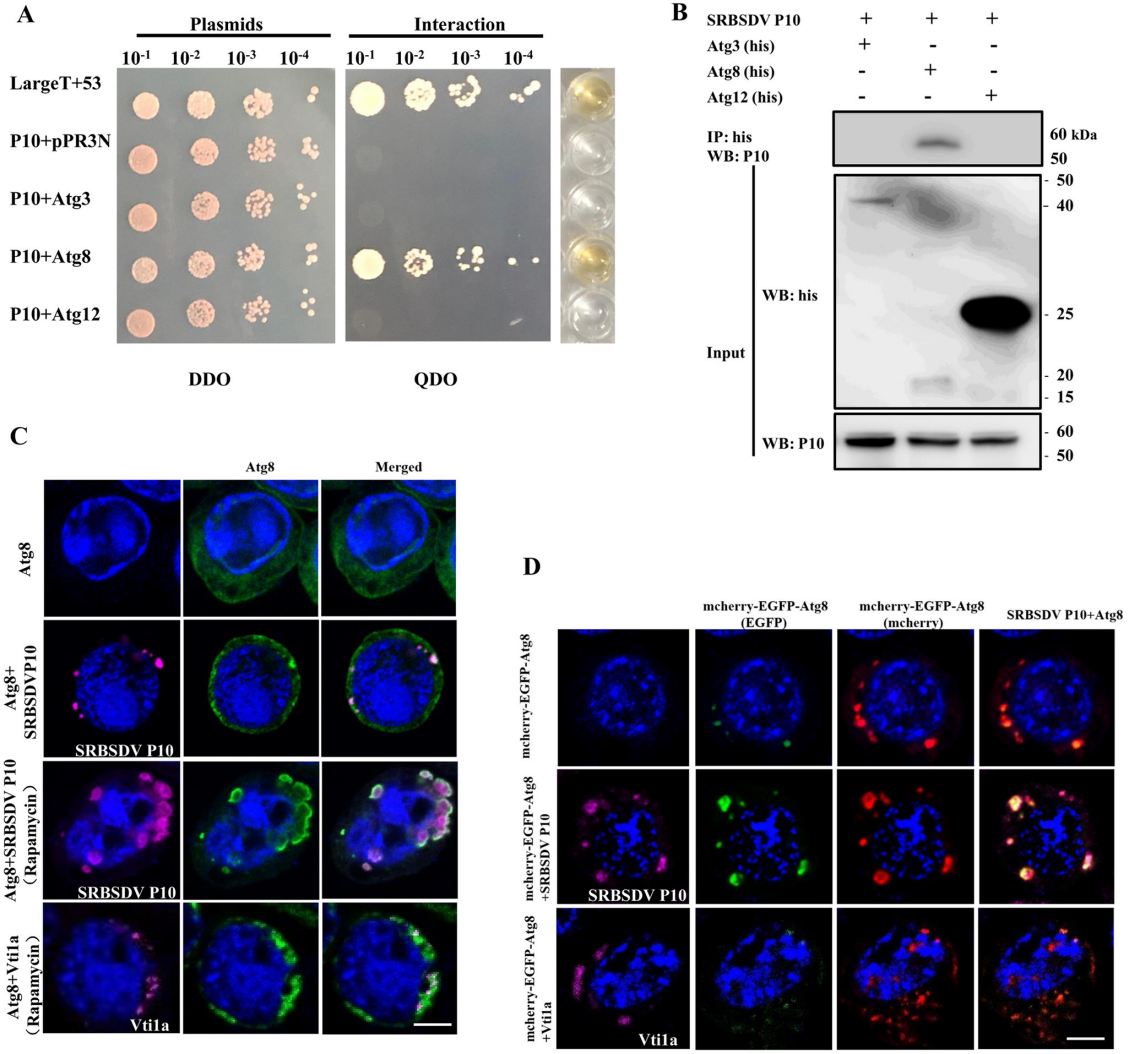
Autophagosome membrane serves as a site for P10 aggregation in cultured Sf9 cells. (**A**) Interaction between SRBSDV P10 and Atg3, Atg8 or Atg12 detected using an *in vivo* yeast two-hybrid assay. Yeast strain NMY51 was cotransformed with all possible pairs of the three proteins. Yeast cells, diluted 10^−1^ to 10^−4^ times, were plated onto DDO (SD-trp-leu) and QDO (SD-trp-leu-his-ade) medium. Clones grown on DDO were selected for β-galactosidase activity assay. Large T + P53 was used as the positive control; P10 + PPR3N served as the negative control. (**B**) Confirmation of the protein interactions by *in vitro* co-immunoprecipitation (Co-IP). Sf9 cells were cotransfected with the respective recombinant bacmids SRBSDV P10 together with Atg3 (his), Atg8 (his) or Atg12 (his) for protein expression. After cells were lysed in lysis buffer, the solution was incubated with anti-his magnetic beads for IP in each group. The anti-SRBSDV P10 antibody was used for western blots to check for interactions. (**C**) LSCM for localization of Atg8 (his tag), colocalization of Atg8 with P10 or Vti1a in Sf9 cells. Sf9 cells were fixed and incubated with anti-His antibody labeled with Dylight 488 (green), anti-SRBSDV P10 antibody labeled with Dylight 633 (magenta) and DAPI (blue). Row 1: cells only overexpressing Atg8; row 2: cells overexpressing Atg8 and SRBSDV P10; row 3: cells overexpressing Atg8 and SRBSDV P10 in the presence of 10 μM rapamycin; row 4: cells overexpressing Atg8 and vti1a in the presence of 10 μM rapamycin. Scale bars, 10 μm. (**D**) Localization of mcherry-EGFP-Atg8, colocalization of mcherry-EGFP-Atg8 with SRBSDV P10 or Vti1a in cultured Sf9 cells in the presence of 10 μM rapamycin. Row 1: cells only overexpressing mcherry-EGFP-Atg8; row 2: cells overexpressing SRBSDV P10 and mcherry-EGFP-Atg8; row 3: cells overexpressing Vti1a and mcherry-EGFP-Atg8. Scale bars, 10 μm.

To further explore the relationship between autophagosomes and SRBSDV propagation, we constructed prey vectors WBPH-Atg3/-Atg8/-Atg12 in a yeast two-hybrid assay to respectively to test for their interactions with SRBSDV P10. The results showed that only Atg8 interacted with SRBSDV P10 (Fig 7A). To further confirm these interaction results between P10 and these three proteins, we constructed baculovirus expression vectors pFastbac1-SRBSDV P10 and pFastHTB-Atg3/-Atg8/-Atg12 (His tag) for transfecting Sf9 cultured cells *in vitro* and tested the interaction using co-immunoprecipitation (Co-IP). These results also indicated that SRBSDV P10 did interact with Atg8 (Fig 7B).

We then used antibodies to localize Atg8 and SRBSDV P10 in Sf9 cells. Cells were fixed 48 h after the baculovirus was added, then incubated with anti-His antibody (green), anti-SRBSDV P10 antibody (magenta) and DAPI (blue), then observed with a confocal laser microscope. When Atg8 was expressed alone, it was distributed diffusely in Sf9 cells. However, when SRBSDV P10 was coexpressed with Atg8, the fluorescence signal from Atg8 aggregated as puncta, and SRBSDV P10 colocalized with Atg8. When rapamycin was added to the culture medium for 8 h, the Atg8-labeled membranes enlarged, and SRBSDV P10 was still distributed along the inside and outside of this membrane system, which meant that the increase of autophagosome membrane provided sufficient places for P10 aggregation. We also expressed a control protein vesicle transport V-SNARE protein (Vti1a) of WBPH in Sf9 cells, which is involved in the formation and fusion of some vesicles. It showed that Vti1a neither enlarged nor colocalized with autophagosome membrane after autophagy activated with rapamycin (Fig 7C).

Interestingly, we also found that P10 inhibited the fusion of autophagosomes and lysosomes by means of the mcherry-EGFP-Atg8 autophagy dual-label system, which has been used to measure the smoothness of fusion between autophagosome and lysosome [39]. Because the enhanced green fluorescent protein (EGFP) is unstable and quickly degraded by lysosomes but mcherry is not, we constructed a pFastHTB-mcherry-EGFP-Atg8 dual fluorescent expression vector and treated Sf9 cells with rapamycin for 8 h to test whether the autophagic flux was obstructed. When autophagosomes fuse with lysosomes, EGFP is degraded, and the autophagosomes fluoresce red. When the binding of autophagosomes to lysosomes is blocked, EGFP cannot be degraded, and the cells fluoresce yellow. The autophagosomes of cells expressing only mcherry-EGFP-Atg8 mostly fluoresced red, and the green signal was weaker. When mcherry-EGFP-Atg8 was coexpressed with SRBSDV P10 or Vti1a, the autophagosomes were yellow and colocalized with P10, but autophagosomes were also mostly fluoresced red in coexpressed with Vti1a (Fig 7D). These results signified that P10 blocked the maturation of autophagosomes to form autolysosomes.

### SRBSDV P10 provided a spatial barrier to inhibit the fusion of autophagosomes and lysosomes

**Fig 8:**
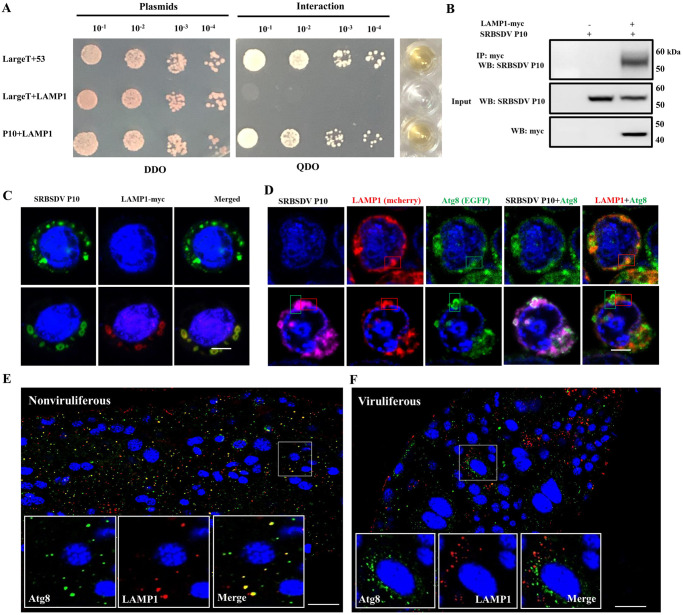
SRBSDV P10 provides a physical barrier to inhibit the fusion of autophagosomes and lysosomes. (**A**) Interaction between SRBSDV P10 and LAMP1 detected using yeast two-hybrid assay. Yeast cells, diluted 10^−1^ to 10^−4^ times, were plated onto DDO (SD-trp-leu) and QDO (SD-trp-leu-his-ade) medium. Clones grown on DDO were selected for β-galactosidase activity assay. Large T + P53 was used as the positive control; Large T + LAMP1 served as the negative control. (**B**) Confirmation of the protein interactions by Co-IP. After cells were lysed in lysis buffer, the solution was incubated with anti-myc magnetic beads for IP. The anti-SRBSDV P10 antibody was used for western blots to check for any interactions. Negative control cells only overexpressed SRBSDV P10. (**C**) Colocalization of SRBSDV P10 and LAMP1 (myc tag). Cultured Sf9 cells were incubated with anti-SRBSDV P10 antibody labeled with Dylight 488 (green), anti-myc antibody labeled with Dylight 549 (red) and DAPI (blue). Negative control: Sf9 cells only overexpressing SRBSDV P10. Scale bars, 10 μm. (**D**) Localization of SRBSDV P10 (magenta), EGFP-Atg8 (green) or LAMP1-mcherry (red) and colocalization of each protein combination. Experimental Sf9 cells overexpressed SRBSDV P10, LAMP1-mcherry and EGFP-Atg8. Negative control: Sf9 cells overexpressing LAMP1-mcherry and EGFP-Atg8. Sf9 cells were fixed and incubated with anti-SRBSDV P10 antibody labeled with Dylight 633. Scale bars, 10 μm. (**E**, **F**) Localization of LAMP1 labled lysosomes and Atg8 labled autophagosome in nonviruliferous and viruliferous WBPH midgut epithelial cells. Insects were treated with rapamycin and the midguts of the two groups were excised and incubated with anti-Atg8 antibody (green) and anti-LAMP1 antibody (red). Scale bars, 20 μm.

To explore the role of P10 in blocking the fusion process between the autophagosome and lysosome, we constructed the prey vector WBPH-LAMP1, a candidate protein for interacting with SRBSDV P10, for the yeast two-hybrid assay to detect any interaction between them. The assay results showed that LAMP1 interacted with SRBSDV P10 (Fig 8A). We also constructed the baculovirus expression vector pFastbac1-LAMP1-myc to coexpress SRBSDV P10 and WBPH LAMP1-myc in Sf9 cells. When SRBSDV P10 was coexpressed with WBPH LAMP1, WBPH LAMP1-myc interacted with P10, as verified by Co-IP (Fig 8B) and colocalized with P10 on the lysosome membrane (Fig 8C). Then pFastbac1-LAMP1-mcherry and pFastbac1- EGFP-Atg8 baculovirus expression vectors were constructed to identify any relationships among P10, WBPH LAMP1 and WBPH Atg8. When only LAMP1-mcherry and EGFP-Atg8 were co-expressed, 82% of the Sf9 cells were signal quench, due that Atg8-labeled autophagosomes and LAMP1-labeled lysosomes fused, and the EGFP fluorescence signal was sensitive in the acidic lysosome environment. However, only 33.1% of the cells were signal quench when the three proteins were expressed. In the signal-quenched LAMP1-mcherry, EGFP-Atg8, and P10 expressed cells, 7.5% of the EGFP-Atg8-labeled autophagosomes colocalized with LAMP1-mcherry labeled lysosomes, which was lower than that in the LAMP1-mcherry and EGFP-Atg8 expressed cells (61.25%) (S4A and S4B Fig). Besides, almost no LAMP1-mcherry and EGFP-Atg8 signal colocalize after SRBSDV P10 (magenta) localized with the respective proteins (Fig 8D). Colocalization of LAMP1 labled lysosomes and Atg8 labled autophagosome in nonviruliferous or viruliferous WBPH midgut epithelial cells was also observed by LSCM. Frequencies of the two groups respectively were 56.65% and 6.6%, which were similar as that in the Sf9 cells expressed without or with P10. These results indicated that P10 interrupted the fusion of autophagosomes and lysosomes. When viruliferous insects were injected with ds*LAMP1*, there was no significant influence on P10 mRNA level (S5A and S5B Fig). These results indicated that the disruption lysosomal function didn’t not affect the viral titer. Thus, the ability of SRBSDV to block lysosomal degradation was crucial for maintaining its propagation and persistence.

## Discussion

The mechanisms used by viruses to exploit autophagy for facilitating viral replication vary considerably [40]. Some viruses modulate lipophagy (lysosomal degradation of lipids) for their replication [33]. Dengue virus infection induces autophagy of lipid cargo, generating lipid droplets for ATP production in mitochondria to provide energy for viral replication [41]. Other viruses use autophagosomes to protect replicating virions from degradation and release them by exocytosis. For example, picornaviruses are nonlytically packaged inside phosphatidylserine-enriched autophagosome-like vesicles, then released for later transmission [42]. Viruses can also reshape intracellular membranes, which decreases the generation of autophagic membrane structures, to avoid degradation. For example, zika virus directly causes the rearrangement of the endoplasmic reticulum (ER) and formation of vesicular clusters, which then serve as sites for viral RNA replication and virion assembly [43,44]. In the present study, we provide new insight into the propagation pathways of a plant reovirus that exploits autophagosomes in its vector insect.

Viral replication of many reoviruses is thought to occur primarily in cytosolic viroplasms. These globular structures are where newly synthesized viral RNAs and progeny single-layer core particles accumulate and double-layer virions are assembled with the outer capsid at the periphery [45–49]. In addition, cellular factors have also been discovered to function during virus replication and virion assembly. For example, some mammalian reoviruses can remodel the ER or cell membrane to form a scaffold to coordinate viral genome replication and assembly [50,51]. The enterotoxin NSP4 of rotavirus, which is a reovirus, also recruits autophagosomal membranes to viroplasms to enhance viral RNA replication [52]. Although SRBSDV was thought to replicate and assemble in viroplasms in a monolayer cell culture of its vector insect [48], our results also showed that RBSDV could be localized to the autophagosome membrane in the midgut epithelial cells of vector insect (Fig 4, S3 Fig). The numerous mature and assembled SRBSDV virions were around the outer membrane of autophagosomes through the outer major capsid protein P10 interacting with Atg8 in the cells (Figs 4 and 7). Inhibition of autophagy, the number of autophagosomes and mature virions decreased, which leading to the decreased of virus titer (Fig 6). Besides, increase of autophagosome membranes provided ample places for P10 aggregation in Sf9 cells (Fig 7C). Thus, the autophagosome membrane might provide as the scaffold that enables the aggregates and assembly of SRBSDV virions. It also explained that why SRBSDV activated autophagy and the virus titer was positively correlated with autophagy level in its vector insect. Thus, besides to replicate in viroplasms, SRBSDV can also exploit the autophagy pathway and hijack autophagosomes for its propagation to enhance virus accumulation, increasing the likelihood that SRBSDV will survive and spread in the vector insect.

Generally, autophagosomes are formed after autophagy being activated, then they fuse with lysosomes to form autolysosomes where lysosomal enzymes digest the contents [53]. However, we found that autophagosomes are almost all bilayers instead of fusing with lysosomes to become autolysosomes when mature and immature virions were distributed around outside the autophagosome membranes (Fig 4A and 4B), suggesting that SRBSDV can efficiently promote autophagosome accumulation and prevent lysosome production. Then we coexpressed SRBSDV P10 and mcherry-EGFP-Atg8 in Sf9 cells, and identified that P10 prevented autophagosomes fusing with lysosomes via autophagy dual-label system (Fig 7D). Besides, we also found that SRBSDV P10 interacted with LAMP1, and interrupted the fusion of autophagosomes and lysosomes to provide a site that enables the aggregates of SRBSDV proteins to assemble (Fig 8). Some viruses can also block the maturation of autophagosomes in different ways to avoid being degraded by the host’s autophagosomes [54–56]. Other viruses disrupt the fusion of autophagosomes with lysosomes by interfering with the function of regulators such as UVRAG, Rubicon, Beclin-1, presenilin-1, and valosin-containing protein, which are all related to autophagosome maturation and degradation [57–60]. The viral phosphoprotein of human parainfluenza virus type 3 binds to a 29-kDa synaptosome-associated protein and inhibits interaction of the 29-kDa protein with syntaxin17, thereby preventing two soluble *N*-ethylmaleimide-sensitive factor-attachment protein receptors (SNAREs) from mediating autophagosome-lysosome fusion [37]. The neuraminidase of influenza A virus H5N1 markedly deglycosylates and degrades LAMPs to induce lysosomal rupture [61,62]. Although different viruses have various ways to manipulate autophagosome maturation, the roles of viral proteins that directly interact with lysosome membranes are still unknown. Our study is the first to demonstrate that a reovirus can block the fusion of autophagosomes and lysosomes by the binding of viral major outer capsid P10 with LAMP1 in the insect vector, which avoid virus degradation by lysosomes and then ultimately sustain the accumulation of autophagosomes for virus propagation (Fig 8).

Our study found that SRBSDV infection can activate autophagy and upregulate the expression of *Atg3*, *Atg5*, *Atg8* and *Atg12* in the insect (Fig 1). The JNK pathway was thought to induce autophagy by directly affecting the target *Atg* genes [30–32]. Thus, we analyzed the phosphorylation level of JNK and found that the JNK pathway was also activated significantly after SRBSDV infection (Fig 2). After nonviruliferous insects were treated with JNK activator or inhibitor, the up- or downregulation of *Atg3*, *Atg5*, *Atg8* and *Atg12* was also positively correlated with the up- or downregulation of the JNK signaling pathway, consistent with the result for viruliferous insects (Figs 1 and 2). Reoviruses are thought to enter the host cell after the virions attach to the membrane-bound receptor integrins via the endocytic pathway [63–65]. Our previous study showed that SRBSDV P10 interacts with integrin β3 of its vector insect [38]. Here, we found that SRBSDV infection increased the level of integrin β3 in the insect (Fig 2A). The extracellular domain of integrins can bind the extracellular matrix, and its cytoplasmic domains bind to the N-terminal of focal adhesion kinase in the JNK pathway [66]. Blocking integrin β1 through an antibody-mediated approach or downregulating the translation of integrin β1 can also suppress JNK signaling [67]. When we inhibited the expression of integrin β3 by dsRNA injection, JNK signaling was also suppressed in insects (Fig 2B). Therefore, these results indicate that SRBSDV seems to enter the epithelial cells by binding integrin β3, which activates the JNK signaling pathway, then JNK directly upregulates the expression of *Atg3*, *Atg5*, *Atg8* and *Atg12* to activate autophagy.

**Fig 9:**
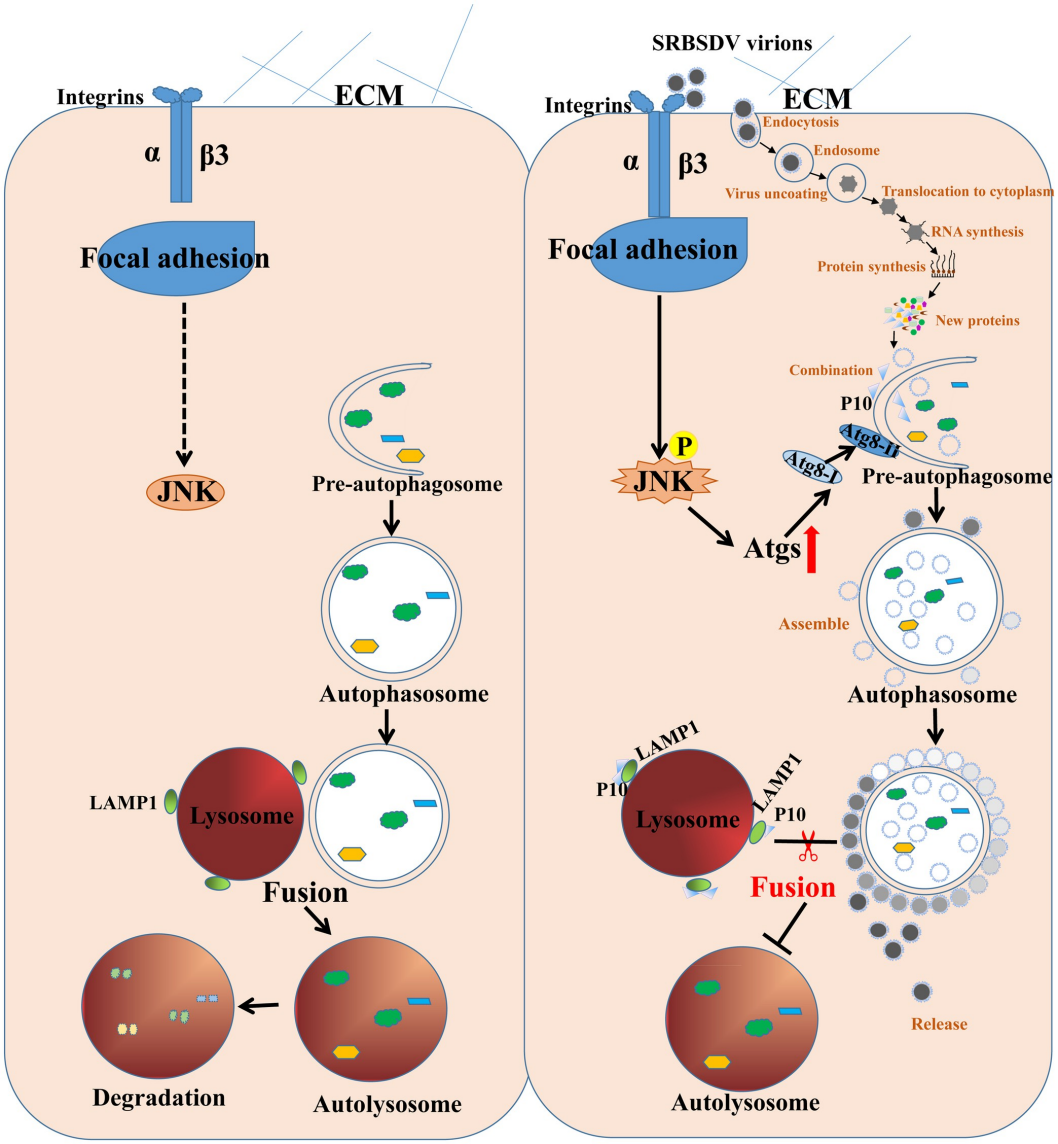
Proposed model for process by which SRBSDV uses outer membrane of autophagosomes for its propagation and virion assembly. In nonviruliferous WBPHs (left side of model), no virions are present to bind with the receptor or activate the JNK pathway and autophagy. Autophagy is at a basal level in insect cells, and autophagosomes ultimately fuse with lysosomes to form autolysosomes for lysosomal degradation. In viruliferous WBPHs (right side), virions bind to the extracellular domain of receptor protein integrin β3 in the cell membrane of the midgut epithelial cells, then the JNK signaling pathway is activated, promoting the expression of autophagy-related (*Atg*) genes and increasing the levels of autophagy by enhancing the formation of Atg8-II. Once the virions enter the cell, they replicate, and new virions gradually assemble on the autophagosome membrane. Viral protein SRBSDV P10 blocks fusion of the autophagosome and lysosome, preventing degradation of the virions by the insect immune system.

Here we propose a model to show how SRBSDV induces an early step in autophagy but inhibits autolysosome formation so that the virus can assembly and accumulation in midgut cells of viruliferous insect (Fig 9). Once the virions enter the midgut lumen during insect feeding, P10 on the virions interacts with the receptor protein integrin β3 on the plasma membrane, activates the JNK signaling pathway in cells, resulting in the upregulated expression of autophagy-related genes and increasing the level of autophagy and number of autophagosomes. After invading the epithelial cells, the virus hijacks the autophagosomal membrane as the site for viral assembly and inhibits the fusion of autophagosomes with lysosomes through the interaction of P10 and LAMP1 in the lysosome membrane to avoid lysosomal degradation.

Recently, a study found that the expression level of *Atg3* and *Atg9* increased after SRBSDV infection the insect, which was consistent with the up-regulation of autophagy gene expression in viruliferous insects found in this study. They also found that inhibition of *Atg3* and *Atg9* could increase the virus titer and speculated autophagy-related genes would functions to suppress SRBSDV propagation, but it was not detected the relationship between autophagy level and virus [68]. These two genes may also affect the accumulation of virus through other pathways. Even the autophagy key gene Atg8 sometimes facilitated virus infection in an autophagy-independent manner [69]. Another recent report found that SRBSDV can trigger mitochondrial autophagy to prevent mitochondria-dependent apoptosis and promote viral propagation in WBPH, which was similar with the discovery in our study that the activation of autophagy can enhance virus propagation, but through a different mechanism [70]. However, another plant reovirus, RBSDV also activates autophagy in its corresponding vector SBPH, which leads to the suppression of virus infection and accumulation [12]. After comparing transmission characteristics of two viruses, acquisition efficiency of SRBSDV by WBPH (more than 80%) was higher than that of RBSDV by SBPH (less than 50%) [8,71]. Once the virus infected insect, the circulative transmission period of SRBSDV is about 8–10 days, compared to 14 days for RBSDV [8,9,72]. The activation of autophagy by SRBSDV probably enhances its propagation in insect, leading to higher host efficiency for acquiring and transmitting the virus, whereas autophagy inhibits RBSDV infection and accumulation. Activation of autophagy also facilitates transmission of plant reovirus RGDV in its insect vector [10]. RGDV uses autophagosomes to carry virions from gut epithelial cells to gut lumen in its insect vector [10], but SRBSDV uses the autophagosome membrane for viral propagation, which enhances virus accumulation in the gut epithelial cells. The incomplete autophagy is beneficial to SRBSDV for virus accumulation and transmission in WBPH. This new finding may explain why the insect is readily acquired SRBSDV and transmitted at higher efficiency than in the case of transmitting RBSDV, which will aid the development of novel antiviral strategies.

## Materials and methods

### Virus maintenance and insect rearing

SRBSDV-infected rice plants were originally provided by Professor Guohui Zhou (South China Agricultural University), Nonviruliferous WBPHs were respectively allowed to feed on the SRBSDV- infected rice plants for 2 d to acquire virus [73]. After 14 d, the viruliferous insects were allowed to feed on rice plants for 2 d to transmit the virus, and the newly infected plants were then grown in a greenhouse [74]. Vector insects were reared in beakers on rice seedlings in incubators (GXZ 380C, China) at 28°C with 16 h light and 8 h dark.

### Primers

Primers used in this study are listed in S5 Table.

### Antibodies and reagents

The anti-SRBSDV P10 antibody was kindly gifted by Professor Jianxiang Wu (Zhejiang University). The other antibodies were purchased: anti-β actin antibody (Proteintech, 66008), anti-P-JNK antibody (Bioss, BS-4163R), anti-Atg8 antibody (Abgent, AP1802a), anti-LAMP1 antibody (Bioss, BSM-51301M), goat anti-mouse IgG+HRP antibody (Solarbio, SE131), anti-Myc tag antibody (Proteintech, 66004), anti-His tag antibody (Cell Signaling Technology, 2365), Dylight 488 goat anti-rabbit IgG (Abbkine, A23220), Dylight 488 goat anti-mouse IgG (Abbkine, A23210), Dylight 549 goat-anti-mouse IgG (Abbkine, A23310), goat anti-mouse IgG+HRP (Thermo Fisher, 32430), goat anti-rabbit IgG+HRP (Thermo Fisher, 32460). Other reagents used and their sources are mounting medium with 4′6-diamidino-2-phenylindole (DAPI) (Abcam, AB104139), rapamycin (Solarbio, IR0010), 3-methyladenine (3-MA) (Solarbio, IM0190), anisomycin (TargetMol, T6758), SP600125 (Abcam, AB120065).

### Recombinant plasmid construction

*Atg3*, *Atg8* and *Atg12* from WBPH were amplified by specific primers and inserted into prey vector pPR3-N (S1 Table). SRBSDV *P10*, *LAMP1* (myc tag), LA*MP1-mcherry*, *EGFP-Atg8* and *mcherry-EGFP-Atg8* were respectively constructed into the baculovirus expression vector pFastBac1 (Invitrogen, 10359–016), and *Atg3*, *Atg8* and *Atg12* were respectively cloned into the pFastBacHTB plasmid (Invitrogen, 10584–027).

### Sf9 cell line incubation and transfection

Sf9 cells were incubated at 27°C in Sf-900 III serum-free medium (Gibco, 12658–019) amended with 5% v/v fetal bovine serum and 1% w/v mycillin. Sf9 cells were transfected using Cellfectin II (Invitrogen, 10362100) according to the protocol of the manufacturer. About 2 × 10^6^ cells were added to each well, incubated for 30 min at 27°C, then a mixture of 2 μg recombinant Bac-plasmids and 8 μl Cellfection II was added to each well and incubated in unsupplemented Grace’s Insect Medium (Thermo Fisher, 11595030) at 27°C for 5 h. Then the transfection mixture was replaced with Sf-900 III serum-free medium to incubate the transfected Sf9 cells at 27°C for 2 d [73].

### Co-immunoprecipitation

The plate with Sf9 cells plus recombinant baculovirus of the prey (protein that interacts with bait) was the negative control, and the experimental cells included bait (protein directly bound to its antibody) and prey [75]. The medium in the two plates was removed after 2 h, then the cells were washed with PBS, Sf-900 III serum-free medium was added, and cells were incubated at 27°C for 48 h. Cells of these experimental group and the negative control were collected at 48 h, then total proteins were extracted with protein extraction buffer (50 mM Tris-HCl, pH 7.5; 150 mM NaCl; 4 mM MgCl_2_; 5 nM DTT; 1% v/v NP-40). The supernatant was added to 50 μl Protein A/G agarose beads (Pierce, 20421), and the mixture was centrifuged to eliminate nonspecifically bound proteins with the beads. After centrifugation, 1 μl of anti-bait antibody was added to bind the bait protein, and the solution was incubated with shaking at 100 rpm and 4°C for 2 h, then 100 μl of beads was added to combine with the anti-bait antibody. The mixture was centrifuged after 2 h, then 1 ml of elution buffer (50 mM Tris-HCl, pH 7.5; 150 mM NaCl; 4 mM MgCl_2_; 2 nM DTT; 1% v/v NP-40) was added to rinse the beads 3 times by centrifugation, and the supernatant was finally discarded. The bead mixture was combined with 200 μl of 2× SDS-PAGE loading buffer and boiled for 10 min. Proteins were separated by SDS-PAGE and transferred to a polyvinylidene fluoride (PVDF) membrane for western blots with anti-prey antibody to detect any interaction.

### Yeast two-hybrid assay

Using the Dualmembrane Pairwise Interaction Kit and included protocol (Dualsystems Biotech, P1501) [74], a clone of yeast strain NMY51 was incubated in 50 ml yeast peptone dextrose adenine agar (YPDA) at 30°C with shaking until the OD546 reached 0.6–0.8. After centrifugation, NMY51 was resuspended in 2.5 ml water. Then 1.5 μg bait and 1.5 μg prey were combined with 100 μl resuspended yeast cells and added to 300 μl PEG/LiOAc Master Mix (50% v/v PEG, 1 M LiOAc and 125 μl single-stranded carrier DNA). After incubation at 42°C for 45 min, the mixture was pelleted for 5 min at 700 × *g*, and the pellet mixed with 150 μl 0.9% w/v NaCl in ddH_2_O, then plated onto selection plates (DDO: SD/ -Leu/ -Trp, QDO: SD/ -Ade/ -His/ -Leu/ -Trp) with 20 mM 3-aminotriazole (3-AT) and incubated for 4 d at 30°C. The strength of the interaction between the bait and prey was evaluated using the Pierce Yeast Beta-galactosidase Assay Kit (Pierce, 75768).

### Inhibition or activation of autophagy or JNK pathway by chemicals

Insects were respectively fed either 1 μM 3-MA or 10 μM rapamycin in 15% w/v sucrose solution for 24 h (to inhibit or activate autophagy, respectively), and the control insects were fed 15% w/v sucrose solution [10]. Nonviruliferous insects were treated with 5 μM anisomycin, or the 50 μM SP600125 to activate or inhibit JNK pathway. Sf9 cells were treated with 20 μM rapamycin (dissolved in PBS) for 8 h, then the solution was replaced with Sf-900 III medium.

### RNA interference assay

The T7 Ribomax Express RNAi System (Promega, P1700) was used to synthesize dsRNAs. Each treatment group included 200 viruliferous third-instar WBPH nymphs, which were injected with 23 nl of either ds*GFP* (3 μg/μl), ds*Atg3*(3 μg/μl), ds*Atg8* (3 μg/μl) or ds*Atg12* (3 μg/μl) using the Drummond Nanoject II Auto-Nanoliter Injector, then reared on healthy rice plants in incubators for 2 d. Total protein was extracted from 50 nymphs and total RNA from 50 other nymphs to analyze the influence of the injections on virus replication by RT-qPCR. Another 100 insects were analyzed to determine the percentage of infected insects by RT-PCR. Viruliferous insects were allowed to feed on rice plants for 2 d, then the plants were allowed to grow in a greenhouse for 14 d, when total RNA of each plant was extracted to detect the *P10* gene by RT-PCR to calculate the transmission efficiency of SRBSDV by each insect. To explore the relationship between integrin β3 and P-JNK after virus acquisition, we injected 100 viruliferous third-instar WBPH nymphs with 23 nl of either ds*GFP* (3 μg/μl) or ds*ITGB3* (3 μg/μl). After nymphs were reared on rice plants, the total protein was extracted from each treatment group in bulk for western blot analysis.

### RT-qPCR

cDNA was synthesized from 1 μg total RNA extracted from WBPH using a FastQuant RT kit with gDNase (TianGen, KR118-02). The RT-qPCR procedure was carried out according to the protocol of the SuperReal PreMix Plus (SYBR Green) kit (GenStar, A304) and a QuantStudio 6 Flex thermocycler (ABI, 100119). Beta-actin was used as the housekeeping gene for normalization in the respective experiments. The mRNA transcript levels were calculated using the formula 2^−ΔΔCt^. The experiments were done three times independently.

### Immunofluorescence microscopy

Sf9 cells that had been previously fixed on cover slips or freshly excised insect tissues were incubated in 4% w/v paraformaldehyde in PBS for 1 h at room temperature and washed three times with PBS. The samples were subsequently permeabilized in 2% v/v Triton X-100 in PBS for 30 min at room temperature and incubated with primary antibody labeled with Dylight 488 (green) or Dylight 549 (red) overnight at 4°C and with DAPI (for Sf9 cells) for 2 h at room temperature [73]. All samples were viewed with LSCM (Zeiss, LSM880), and the images were saved using ZEN Blue 2011.

### Electron microscopy and immunoelectron microscopy

For electron microscopy, excised midguts of the insects were fixed in 2% w/v paraformaldehyde with 2% w/v osmium tetroxide in PBS for 2 h, dehydated in an ethanol series (30%, 50%, 70%, 90%, 95% and 100%), then embedded in LR Gold Resin (Sigma, 62659). The specimens were sectioned with an ultramicrotome (Leica, EM UC7), then picked up on 200-mesh copper grids. The sections were respectively stained using 2% w/v uranyl acetate (in 50% ethanol) and alkaline lead citrate [0.08 M Pb (NO_3_)_2_; 0.12 M C_6_H_5_Na_3_O_7_·2H_2_O] for 5 to 10 min.

For immunoelectron microscopy, excised midguts or Sf9 cells were fixed in 4% v/v paraformaldehyde in PBS at 4°C for 6 h, dehydated in an ethanol series (30%, 50%, 70%, 90%, 95% and 100%), then embedded in LR White Resin (Sigma-Aldrich, 62662). The specimens were picked up on 200-mesh nickel grids coated with formvar–carbon. Then they were incubated for 30 min in blocking buffer, then at room temperature with primary antibody for 1.5 h, followed by 3 washes and incubation with 5-nm- and 10-nm-diameter gold-conjugated secondary antibodies for 1.5 h. After 3 more washes, they were then stained. The sections were examined with a transmission electron microscope (FEI, TECNAI G2 F20) at 80-kV accelerating voltage.

## Supporting information

S1 Fig Inhibition of integrin β3 during the process of virus entering in the midgut epithelial cells decrease the relative mRNA level of *Atgs*. **A** Relative mRNA level of integrin β3 after ds*GFP* or ds*ITGB3* injection as determined by RT-qPCR. **B** Relative mRNA level of *Atgs* in WBPHs after ds*GFP* or ds*ITGB3* injection. The data was obtained from twenty insects in each group (*P < 0.05, **P < 0.01). C Protein expression level of *Atg*s after ds*GFP* or ds*ITGB3* injection.
(TIF)

S2 Fig TEM of nonviruliferous vectors midgut epithelial cells. Midgut epithelial cells of nonviruliferous WBPH. Images on right are closeups of the respective boxed areas to the left. N: cell nucleus. Scale bars, 500 nm.
(TIF)

S3 Fig Steps in the process of autophagosomes engulfing SRBSDV virions. **A**, **B** Attachment of immature SRBSDV virions on autophagy-like bilayered membranes. Black arrow: immature SRBSDV virions. **C**, **D** Immature SRBSDV virions become engulfed by pre-autophagosomes. **E** Maturation of pre-autophagosome, which has engulfed immature SRBSDV virions. **A**-**E**: Scale bars, 200 nm.
(TIF)

S4 Fig Quantitive analyses on colocalizations of lysosomes and autophagosomes. **A** Percentage of Sf9 cells that were signal quench when the cells coexpressed LAMP1-mcherry and EGFP-Atg8 together with or without SRBSDV P10. **B** Frequency of colocalization of LAMP1-mcherry and EGFP-Atg8 when the cells expressed LAMP1-mcherry and EGFP-Atg8 together without or with SRBSDV P10. **C** Frequency of colocalization of LAMP1 labled lysosomes and Atg8 labled autophagosome in nonviruliferous and viruliferous WBPH midgut epithelial cells. (*P < 0.05, **P < 0.01, Student’s t-test).
(TIF)

S5 Fig Inhibition of LAMP1 did not change mRNA level of P10 in SRBSDV-viruliferous WBPHs. **A** Relative mRNA level of LAMP1 in SRBSDV-viruliferous WBPHs after ds*GFP* or ds*LAMP1* injection as determined by RT-qPCR. **B** Relative mRNA level of P10 in WBPHs after ds*GFP* or ds*LAMP1* injection. (Means ± SEM of three independent experiments *P < 0.05, **P < 0.01).
(TIF)

S1 Table Number of SRBSDV infected WBPHs after feeding on 15% w/v sucrose solution, 1 μM 3-MA and 10 μM rapamycin. (XLSX)

S2 Table SRBSDV transmission efficiency by viruliferous WBPHs fed 15% w/v sucrose solution, 1 μM 3-MA and 10 μM rapamycin. (XLSX)

S3 Table Number of SRBSDV-infected WBPHs after injection with ds*GFP*, ds*Atg3*, ds*Atg8* or ds*Atg12*. (XLSX)

S4 Table SRBSDV transmission efficiency by viruliferous WBPHs injected with ds*GFP*, ds*Atg3*, ds*Atg8* or ds*Atg12*. (XLSX)

S5 Table Primers used in this study. (XLSX)
