# Supplementary figures and images for: Southern rice black-streaked dwarf virus induces incomplete autophagy for persistence in gut epithelial cells of its vector insect
Source: PLoS Pathog. 2023 Jan 27;19(1):e1011134. doi: 10.1371/journal.ppat.1011134 (PMC9907856; doi:10.1371/journal.ppat.1011134)

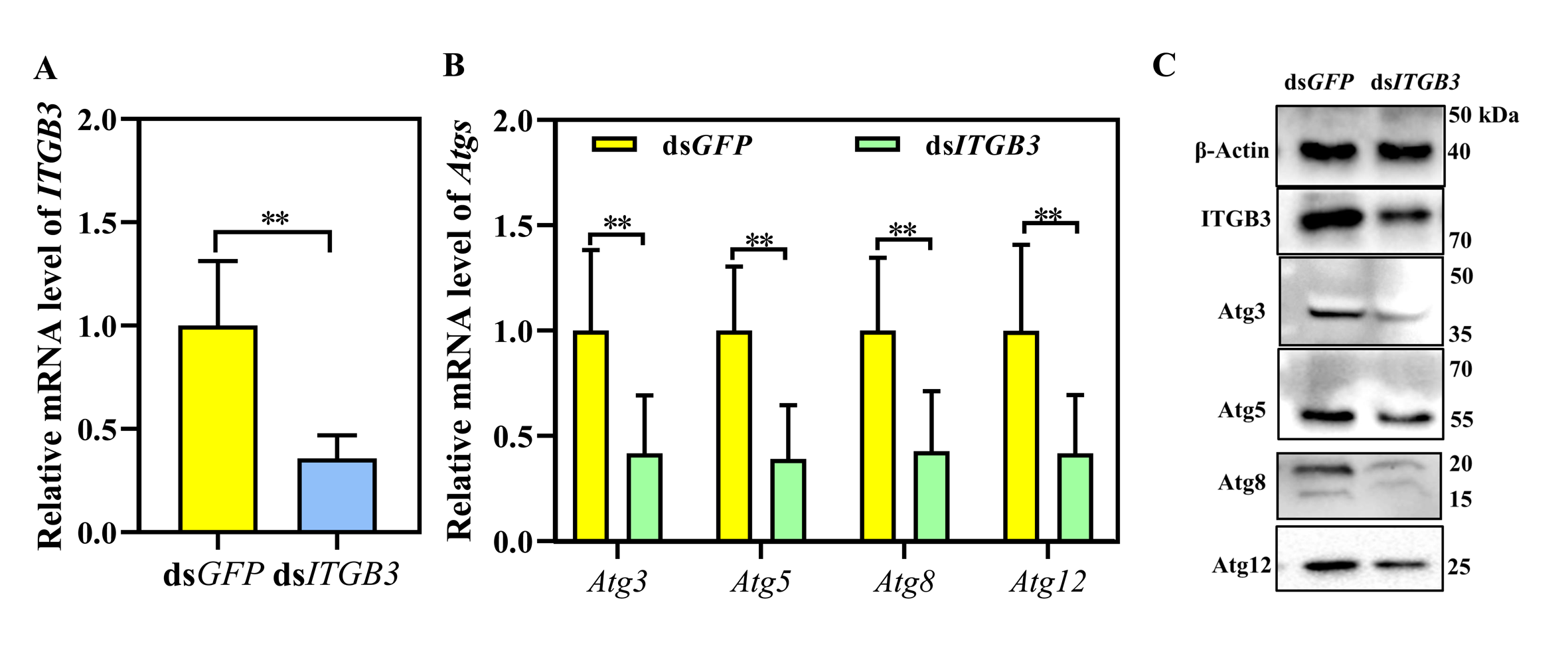

Supplement: S1 Fig — A Relative mRNA level of integrin β3 after dsGFP or dsITGB3 injection as determined by RT-qPCR. B Relative mRNA level of Atgs in WBPHs after dsGFP or dsITGB3 injection. The data was obtained from twenty insects in each group (*P < 0.05, **P < 0.01). C Protein expression level of Atgs after dsGFP or dsITGB3 injection. (TIF) [file ppat.1011134.s001.tif]

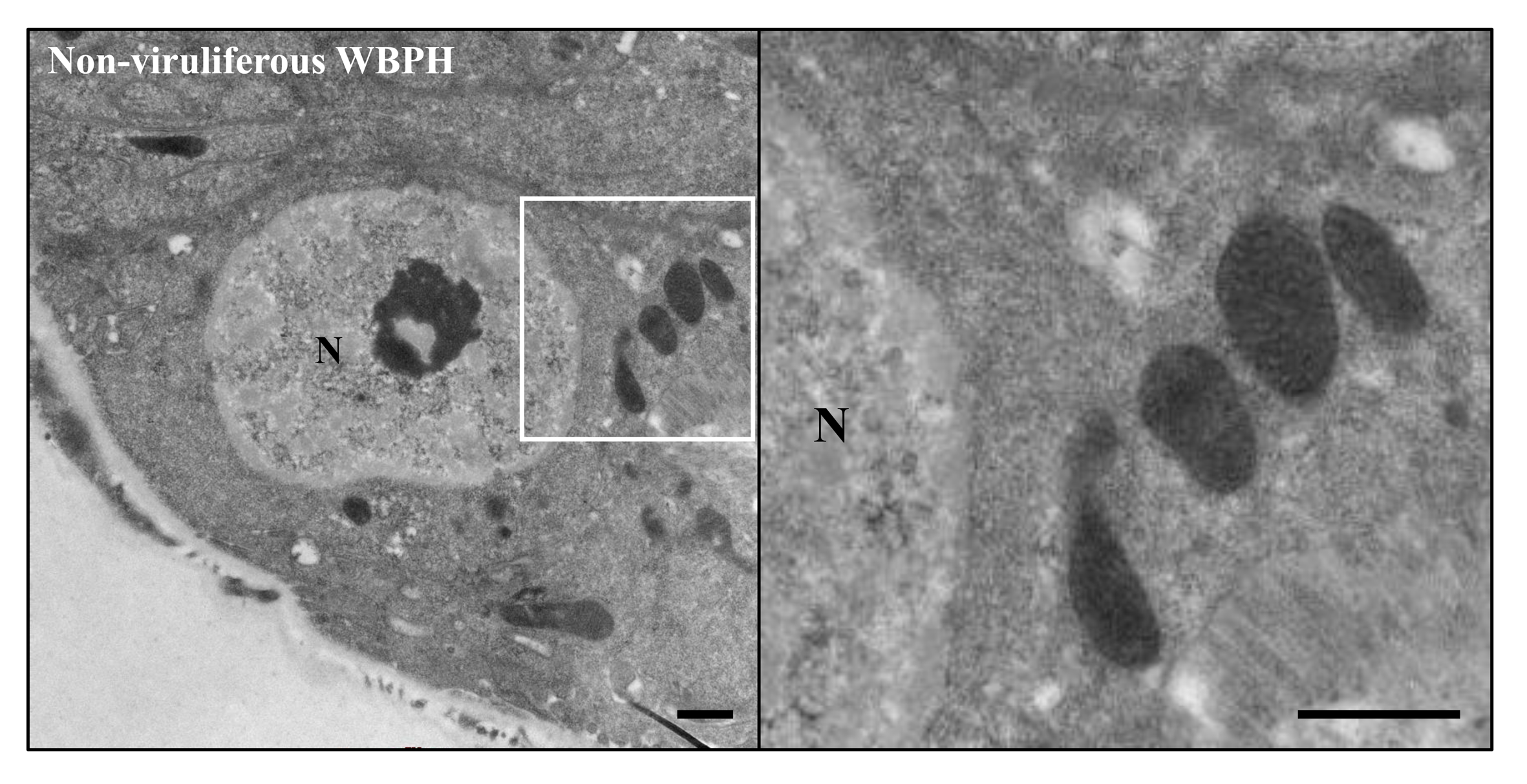

Supplement: S2 Fig — Midgut epithelial cells of nonviruliferous WBPH. Images on right are closeups of the respective boxed areas to the left. N: cell nucleus. Scale bars, 500 nm. (TIF) [file ppat.1011134.s002.tif]

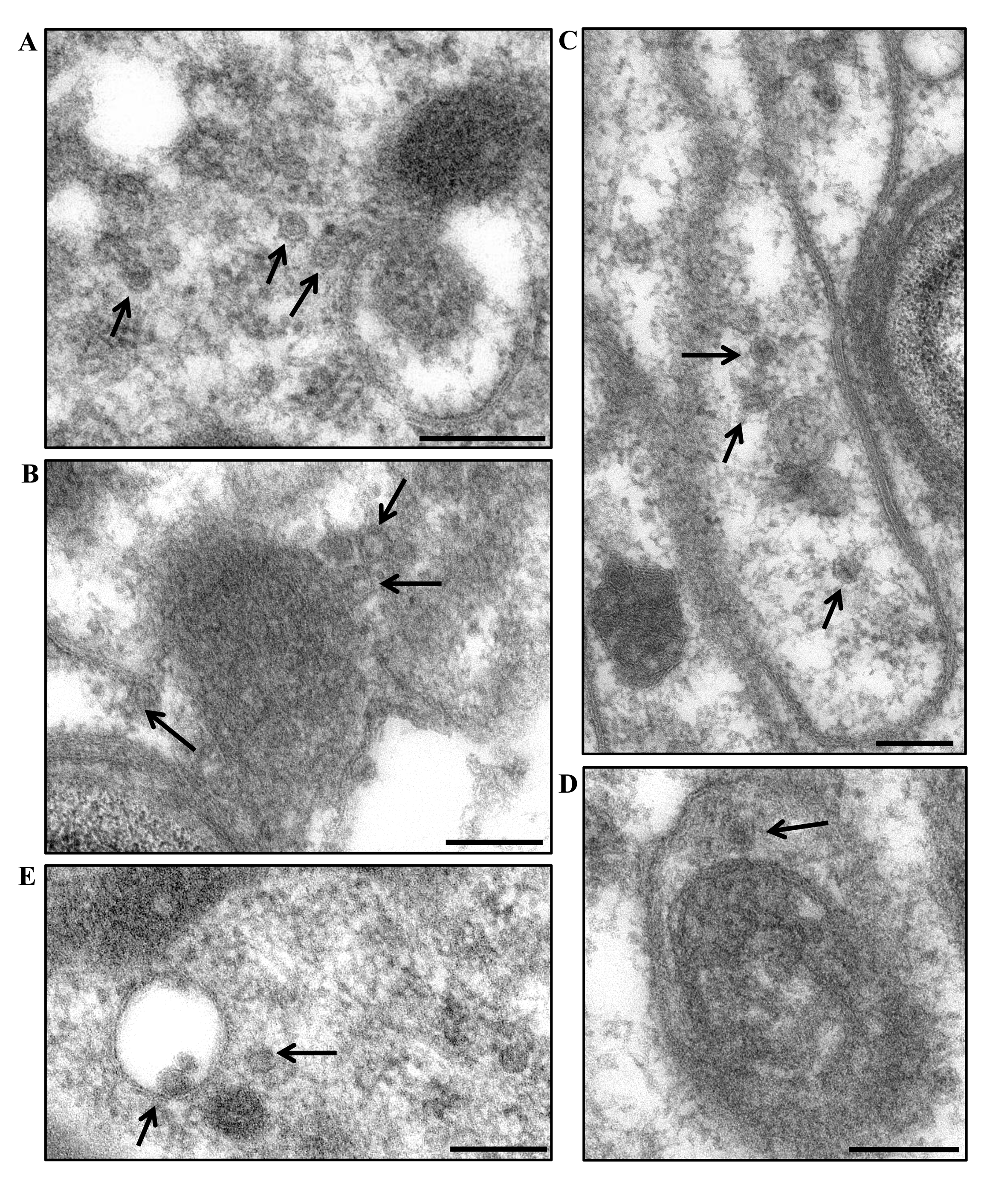

Supplement: S3 Fig — A, B Attachment of immature SRBSDV virions on autophagy-like bilayered membranes. Black arrow: immature SRBSDV virions. C, D Immature SRBSDV virions become engulfed by pre-autophagosomes. E Maturation of pre-autophagosome, which has engulfed immature SRBSDV virions. A-E: Scale bars, 200 nm. (TIF) [file ppat.1011134.s003.tif]

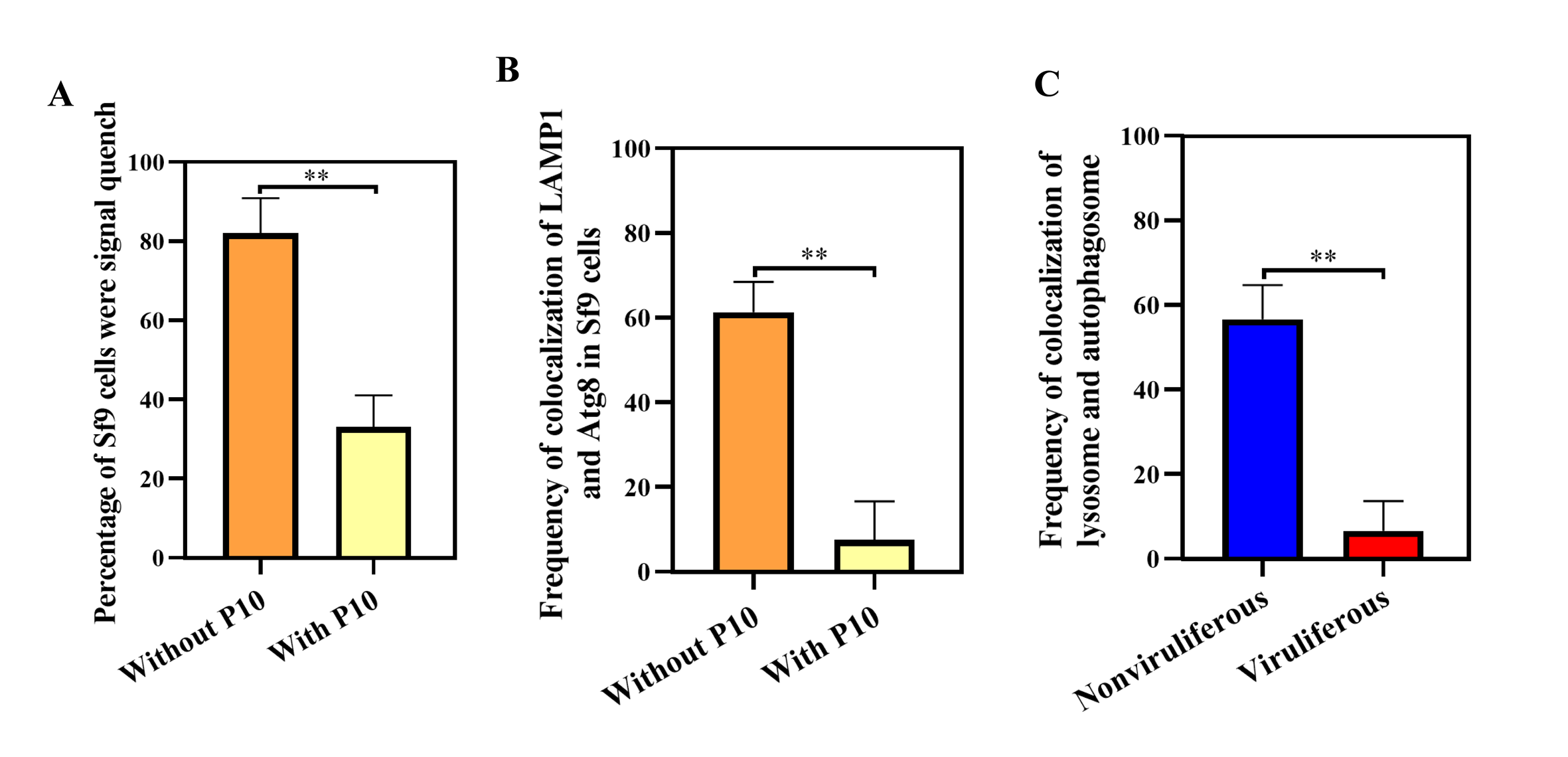

Supplement: S4 Fig — A Percentage of Sf9 cells that were signal quench when the cells coexpressed LAMP1-mcherry and EGFP-Atg8 together with or without SRBSDV P10. B Frequency of colocalization of LAMP1-mcherry and EGFP-Atg8 when the cells expressed LAMP1-mcherry and EGFP-Atg8 together without or with SRBSDV P10. C Frequency of colocalization of LAMP1 labled lysosomes and Atg8 labled autophagosome in nonviruliferous and viruliferous WBPH midgut epithelial cells. (*P < 0.05, **P < 0.01, Student’s t-test). (TIF) [file ppat.1011134.s004.tif]

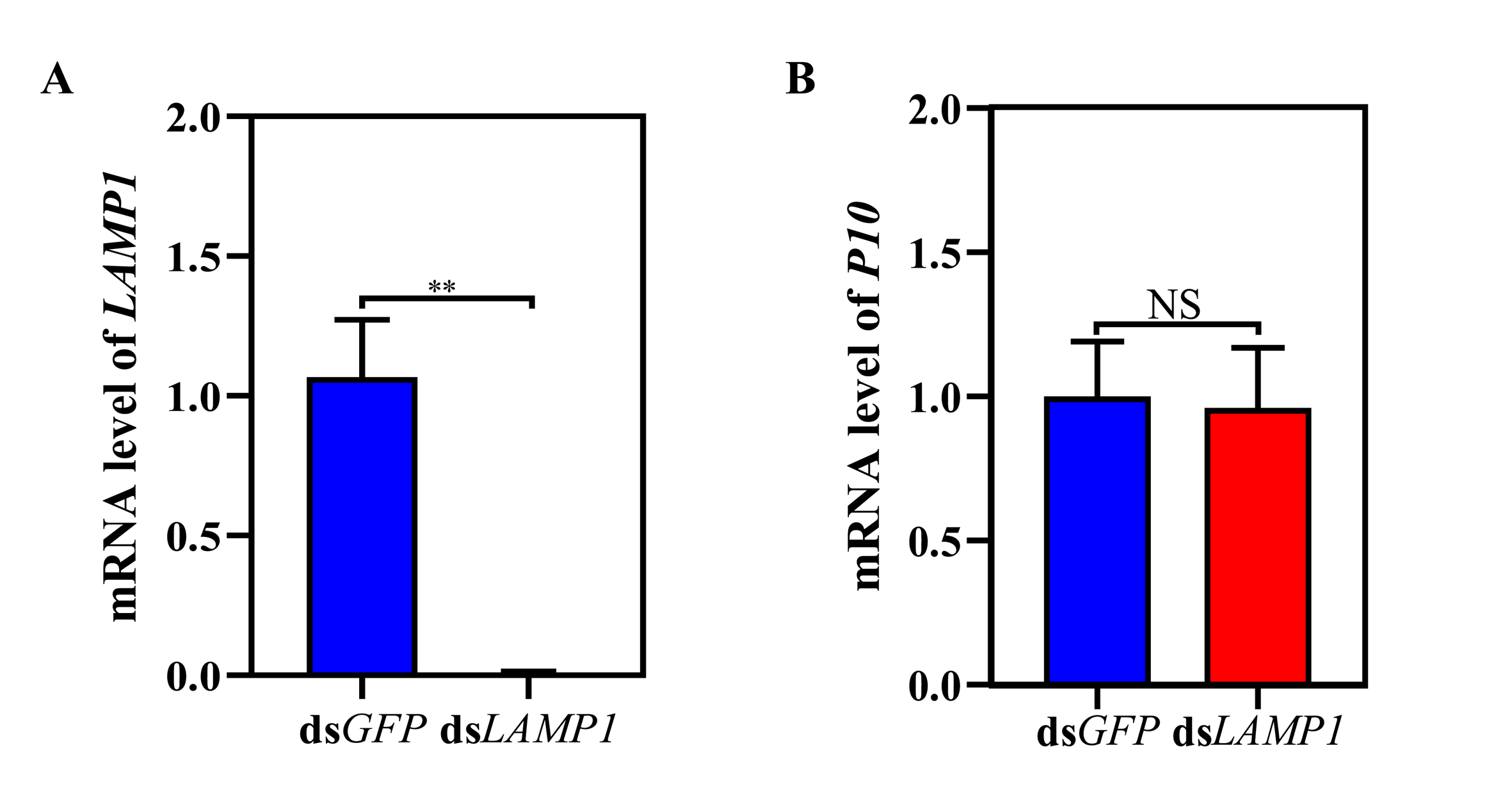

Supplement: S5 Fig — A Relative mRNA level of LAMP1 in SRBSDV-viruliferous WBPHs after dsGFP or dsLAMP1 injection as determined by RT-qPCR. B Relative mRNA level of P10 in WBPHs after dsGFP or dsLAMP1 injection. (Means ± SEM of three independent experiments *P < 0.05, **P < 0.01). (TIF) [file ppat.1011134.s005.tif]
